# First thia-Diels–Alder reactions of thiochalcones with 1,4-quinones

**DOI:** 10.3762/bjoc.14.156

**Published:** 2018-07-19

**Authors:** Grzegorz Mlostoń, Katarzyna Urbaniak, Paweł Urbaniak, Anna Marko, Anthony Linden, Heinz Heimgartner

**Affiliations:** 1 Department of Organic and Applied Chemistry, University of Łódź, Tamka 12, PL 91-403 Łódź, Poland; 2 Department of Inorganic and Analytical Chemistry, University of Łódź, Tamka 12, PL 91-403 Łódź, Poland; 3 Department of Chemistry, University of Zurich, Winterthurerstrasse 190, CH-8057 Zurich, Switzerland

**Keywords:** hetero-Diels–Alder reactions, quinone dyes, quinones, sulfur heterocycles, thiochalcones

## Abstract

Aryl and hetaryl thiochalcones react smoothly with 1,4-quinones in THF solution at 60 °C yielding the corresponding fused 4*H*-thiopyrans after spontaneous dehydrogenation of the initially formed [4 + 2] cycloadducts. In general, the yields of the isolated products were high. With 5-chloro-10-hydroxy-1,4-anthraquinone, the thia-Diels–Alder reaction occurred with complete regioselectivity. In the case of the reaction of vitamin K_3_ (menadione) with diphenylthiochalcone, the initial cycloadduct was isolated in 37% yield.

## Introduction

Hetero-Diels–Alder reactions are considered to be a powerful methodology widely explored for the synthesis of six-membered heterocycles [1–2] with numerous applications for the construction of complex molecules including naturally occurring products [3–4], drugs [5–6], agrochemicals [7], etc. In addition, asymmetric hetero-Diels–Alder reactions are of current interest [8–10]. Whereas aza- and oxa-Diels–Alder reactions are frequently applied, thia-Diels–Alder reactions are rarely reported. However, aryl and hetaryl thioketones are known to react as ‘superdienophiles’, thereby yielding the corresponding 3,6-dihydro-2*H*-thiopyrans [11–14].

Despite the fact that thiochalcones exist in solution as mixtures of dimers [15–16], they enter into cycloaddition reactions not only as heterodienes [17–19], but also as heterodipolarophiles [15]. In two recent publications we reported new thia-Diels–Alder reactions of aryl, hetaryl and ferrocenyl-substituted thiochalcones with acetylenic dienophiles, which lead to the corresponding 4*H*-thiopyrans in a regioselective manner [16,20].

In cycloaddition chemistry, 1,4-quinones are applied widely both as dipolarophiles and dienophiles. In the case of [3 + 2] cycloadditions, reactions can occur chemoselectively with either the C=O or the C=C unit [21–23]. On the other hand, reactions with diverse 1,3-dienes and heterodienes generally occur at the C=C group [24–26]. To the best of our knowledge, thia-Diels–Alder reactions of 1,4-quinones with thiochalcones have not yet been reported.

In the present study, thia-Diels–Alder reactions of aryl and hetaryl thiochalcones with selected 1,4-quinones, such as 1,4-benzoquinone, 1,4-naphthoquinone, and 1,4-anthraquinone were investigated as a route to novel 4*H*-thiochromene-5,8-dione derivatives.

## Results and Discussion

Aryl and hetary lthiochalcones **1a**–**d** are easily obtained by treatment of the corresponding chalcones with Lawesson′s reagent in THF solution [15]. Along with the commercially available 1,4-benzoquinone (**2a**) and 1,4-naphthoquinone (**2b**), 1,4-anthraquinone (**2c**) and 5-chloro-10-hydroxy-1,4-anthraquinone (**2d**) were prepared from quinizarine according to known procedures [27–28].

**Scheme 1:**
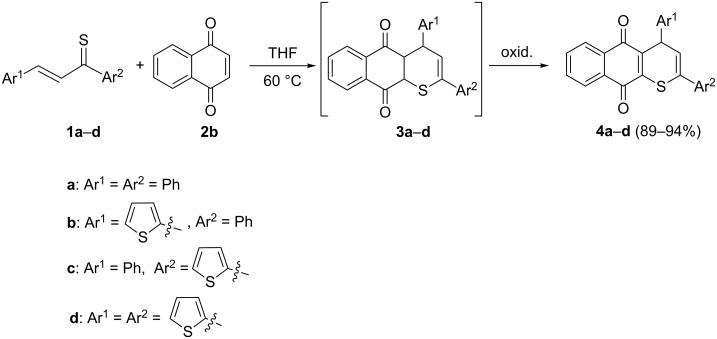
Reactions of aryl/hetarylthiochalcones **1a**–**d** with 1,4-naphthoquinone (**2b**).

First experiments were performed with **2b** and thiochalcones **1a**–**d** in THF solutions at 60 °C by starting with equimolar amounts of substrates. After 2 h, completion of the reaction was confirmed by TLC, and, after typical workup, products **4** were obtained as colored solids in high yields (Scheme 1).

The ^1^H NMR analysis revealed that the initially formed [4 + 2] cycloadducts **3** underwent spontaneous oxidation under the reaction conditions. The structures of type **4** were confirmed by spectroscopic methods and elemental analysis. For example, the ^1^H NMR spectrum of **4a** showed two doublets at 5.46 and 6.35 ppm with *J* = 6.4 Hz attributed to H–C(4) and H–C(3), respectively. In the ^13^C NMR spectrum, the sp^3^-C(4) atom absorbs at 40.9 ppm and the signals for the two C=O groups were found at 180.8 and 181.4 ppm, respectively.

**Scheme 2:**
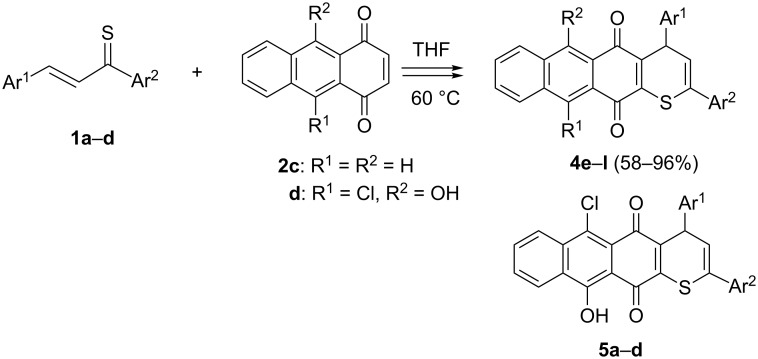
Reactions of thiochalcones **1a**–**d** with 1,4-anthraquinones **2c** and **2d**.

In the second series of experiments, thiochalcones **1a**–**d** were subjected to reaction with the symmetrical 1,4-anthraquinone (**2c**) and its non-symmetrically substituted derivative **2d**. In analogy to the reactions with **2b**, the expected products **4e**–**h** were isolated in all cases as stable colored solids in high yields (Scheme 2).

**Figure 1:**
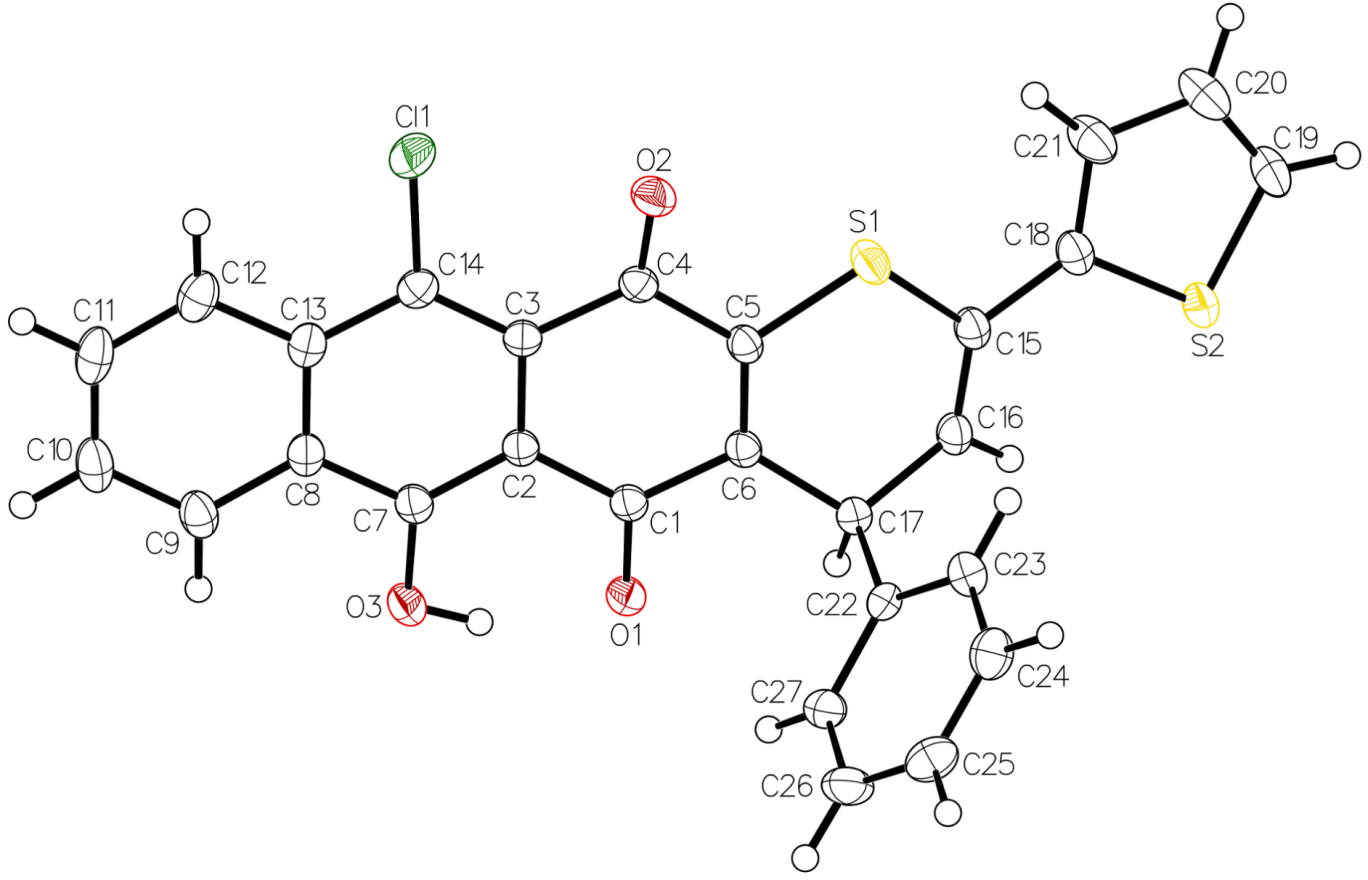
ORTEP plot [29] of the molecular structure of **4k** showing the major conformation of the disordered thiophene ring (50% probability ellipsoids; arbitrary numbering of the atoms).

The reactions of thiochalcones **1a**–**d** with **2d** require a brief discussion. In these cases, the formation of two regioisomeric cycloadducts could be expected, but the ^1^H NMR analysis of the crude products showed that only one product was present in each case and, therefore, the studied [4 + 2] cycloaddition reactions occurred with complete regioselectivity. Based on the assumption that the nucleophilic S-atom of the thiochalcone attacks the more electrophilic C-atom, we postulate that compounds **4i**–**l** and not their isomers **5a**–**d** are formed in these reactions. This assumption is supported by the intramolecular H-bonding, which enhances the electrophilicity of C(3) in the dienophile **2d**. Finally, the structure of **4k** was established by X-ray crystallography (Figure 1). Remarkably, the presence of OH and Cl substituents in products **4i**–**l** results in a bathochromic shift of UV–vis absorptions.

The crystal structure of **4k** is that of the regioisomer proposed on the basis of reactivity considerations. Since the space group is centrosymmetric, the compound in the crystal is racemic. The S-atom of the thiophene ring is disordered over two unequally occupied positions as a result of slight but opposite directions of envelope puckering of the ring. The hydroxy group forms an intramolecular hydrogen bond with the adjacent quinoid O-atom.

**Figure 2:**
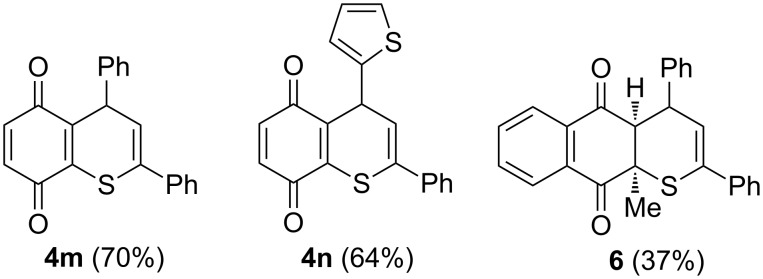
Products of the reactions of thiochalcones **1a** and **1b** with 1,4-benzoquinone (**2a**) and of **1a** with menadione (**2e**).

In addition to anthraquinones **2b** and **2c**, the simple 1,4-benzoquinone (**2a**) was also tested in the reaction with thiochalcones **1**. The reactions performed with **1a** and **1b** delivered the expected 4*H*-thiochromene-5,8-diones **4m**,**n**, which were isolated in good yields using flash chromatography, but underwent decomposition under ambient conditions, and none of them could be obtained in analytically pure form (Figure 2).

Vitamin K_3_ (**2e**, menadione), which is an important representative of 1,4-naphtoquinones, was also involved in the present study and tested in the reaction with diphenylthiochalcone (**1a**). In that case, however, a longer reaction time was required, and substantial amounts of decomposition products were formed. The chromatographic workup led to a brownish fraction, containing a single product, which was identified as the [4 + 2] cycloadduct **6** (Figure 2). Its structure was elucidated by ^1^H NMR spectroscopy, which evidenced the presence of two doublets at 3.76 (*J* = 4.8 Hz) and 6.50 ppm (*J* = 4.3 Hz) and a triplet-like signal at 4.18 ppm with *J* ≈ 4.5 Hz. These signals were attributed to HC(4a), HC(3) and HC(4), respectively. All attempts to prepare an analytically pure sample were unsuccessful. In contrast to all other initially formed cycloadducts, further oxidative conversion to the quinone structure was not possible in this case.

## Conclusion

The present study demonstrates that 1,4-quinones are prone dienophiles in reactions with aryl/hetarylthiochalcones. These thia-Diels–Alder reactions led to a new class of 4*H*-thiopyran derivatives. The presence of a quinone system is an important structural aspect of this class as it is common in many naturally occurring compounds, e.g., dyes such as alizarin, carminic acid, and isoprenoid dyes, as well as drugs such as doxorubicin. In addition, the presence of thiophenyl substituents may be of importance for their applications in materials chemistry.

## Experimental

**General information:** Solvents and chemicals were purchased and used as received without further purification. Products were purified by standard column chromatography on silica gel (230–400 mesh, Merck). Unless stated otherwise, yields refer to analytically pure samples. NMR spectra were recorded with a Bruker Avance III 600 MHz instrument (^1^H NMR: 600 MHz; ^13^C NMR: 151 MHz). Chemical shifts are reported relative to solvent residual peaks (^1^H NMR: δ = 7.26 ppm [CHCl_3_]; ^13^C NMR: δ = 77.0 ppm [CDCl_3_]). IR spectra were registered with a FTIR NEXUS spectrometer (as film or KBr pellets). UV–vis spectra were recorded using a UV–vis JASCO V-630 spectrophotometer. Melting points were determined in capillaries with a Stuart SMP30 apparatus with automatic temperature monitoring.

**Starting materials:** 1,4-Benzoquinone (**2a**) and 1,4-naphthoquinone (**2b**) were commercial reagents and used without further purification. 1,4-Anthraquinone (**2c**) was prepared from commercial quinizarine by treatment with sodium borohydride according to a literature procedure [27]. 5-Chloro-10-hydroxy-1,4-anthraquinone (**2d**) was obtained by treatment of quinizarine with thionyl chloride according to the protocol described in [27]. Thiochalcones **1a**–**d** were prepared according to our protocol reported in an earlier publication [16].

**General procedure:** A solution of 1 mmol of the corresponding thiochalcone **1** and 1 mmol of the 1,4-quinone **2** in 1 mL of dry THF was placed in a thick-walled glass tube, which was closed with a screw cap. The mixtures were heated at 60 °C for 2 h (for **4a**–**n**) or 48 h (for **6**). In the last case, progress of the reaction was monitored by TLC, and an additional amount of thiochalcone **1a** was added in small portions until menadione (**2e**) was completely consumed. The solvent was evaporated in vacuo and the crude mixtures were analyzed by ^1^H NMR first and subsequently purified by flash chromatography using dichloromethane (for **4a**–**n**) or a mixture of petroleum ether and dichloromethane 1:1 (for **6**) as the eluents. For products **4a**–**l**, analytically pure samples were obtained by crystallization from petroleum ether with a small amount of dichloromethane. All attempts to obtain analytically pure samples for **4m**, **4n** and **6** were unsuccessful and the purification procedure led to formation of some decomposition products.

**2,4-Diphenyl-4*H*-benzo[*g*]thiochromene-5,10-dione (4a):** Yield: 340 mg (89%). Red-orange crystals; mp 165 °C (dec.); ^1^H NMR δ 5.46 (d, *J*_H,H_ = 6.4 Hz, Ph-C*H*), 6.35 (d, *J*_H,H_ = 6.4 Hz, C=C*H*), 7.24–7.25 (m, 1CH_arom_), 7.32–7.34 (m, 2CH_arom_), 7.41–7.43 (m, 3CH_arom_), 7.52–7.54 (m, 2CH_arom_), 7.61–7.62 (m, 2CH_arom_), 7.70–7.74 (m, 2CH_arom_), 8.10 (dd, *J*_H,H_ = 7.4 Hz, *J*_H,H_ = 1.2 Hz, 1CH_arom_), 8.13 (dd, *J*_H,H_ = 7.4 Hz, *J*_H,H_ = 1.2 Hz, 1CH_arom_) ppm; ^13^C NMR δ 40.9 (Ph-*C*H), 121.0, 126.6, 126.7, 127.0, 127.5, 128.4, 128.8, 128.9, 129.0, 133.4, 134.3 (14CH_arom_, C=*C*H), 131.7, 132.0, 132.2, 136.4, 137.4, 142.3, 144.54 (6C_arom_, *C*=CH), 180.8, 181.4 (2C=O) ppm; IR ν: 3060 (w), 3028 (w), 1653 (vs, 2C=O), 1591 (s), 1562 (m), 1489 (m), 1451 (m), 1334 (m), 1288 (vs), 1152 (m), 1106 (w), 1030 (m), 913 (m), 834 (m), 710 (s), 694 (s) cm^−1^; UV–vis (CH_2_Cl_2_) λ_max_/nm (lg ε): 243 (4.49), 332 (3.57), 478 (328); anal. calcd for C_25_H_16_O_2_S (380.46): C, 78.92; H, 4.24; S, 8.43; found: C, 78.87; H, 4.24; S, 8.37.

**2-Phenyl-4-(thiophen-2-yl)-4*H*-benzo[*g*]thiochromene-5,10-dione (4b):** Yield: 365 mg (94%). Red-orange crystals; mp 168 °C (dec.); ^1^H NMR δ 5.80 (d, *J*_H,H_ = 6.7 Hz, thiophen-2-yl-C*H*), 6.40 (d, *J*_H,H_ = 6.7 Hz, C=C*H*), 6.95 (dd, *J*_H,H_ = 5.0 Hz, *J*_H,H_ = 3.7 Hz, 1CH_arom_), 7.06 (d, *J*_H,H_ = 3.5 Hz, 1CH_arom_), 7.19 (dd, *J*_H,H_ = 5.0 Hz, *J*_H,H_ = 1.0 Hz, 1CH_arom_), 7.42–7.46 (m, 3CH_arom_), 7.63–7.65 (m, 2CH_arom_), 7.71–7.78 (m, 2CH_arom_), 8.13 (dd, *J*_H,H_ = 7.6 Hz, *J*_H,H_ = 1.1 Hz, 1CH_arom_), 8.18 (dd, *J*_H,H_ = 7.6 Hz, *J*_H,H_ = 1.1 Hz, 1CH_arom_) ppm; ^13^C NMR δ 35.2 (thiophen-2-yl-*C*H), 119.9, 125.3, 125.6, 126.7, 127.0, 127.1, 127.2, 128.8, 129.2, 133.5, 134.4 (12CH_arom_, C=*C*H), 128.5, 131.8, 132.1, 135.3, 137.2, 144.2, 144.3 (6C_arom_, *C*=CH), 180.6, 181.4 (2C=O) ppm; IR ν: 3075 (w), 3031 (w), 1652 (vs, 2C=O), 1589 (s), 1571 (s), 1489 (m), 1442 (m), 1420 (w), 1331 (m), 1289 (vs), 1220 (m), 1151 (m), 1103 (w), 1033 (w), 919 (w), 831 (m), 767 (m), 704 (s), 695 (s) cm^−1^; UV–vis (CH_2_Cl_2_) λ_max_/nm (lg ε): 244 (4.50), 338 (3.59), 463 (3.34); anal. calcd for C_23_H_14_O_2_S_2_ (386.49): C, 71.48; H, 3.65; S, 16.59; found: C, 71.48; H, 3.65; S 16.59.

**4-Phenyl-2-(thiophen-2-yl)-4*H*-benzo[*g*]thiochromene-5,10-dione (4c):** Yield: 355 mg (92%). Red-orange crystals; mp 158 °C (dec.); ^1^H NMR δ 5.42 (d, *J*_H,H_ = 6.5 Hz, Ph-C*H*), 6.41 (d, *J*_H,H_ = 6.5 Hz, C=C*H*), 7.01 (dd, *J*_H,H_ = 5.2 Hz, *J*_H,H_ = 3.4 Hz, 1CH_arom_), 7.23–7.25 (m, 1CH_arom_), 7.28–7.33 (m, 3CH_arom_), 7.36 (dd, *J*_H,H_ = 3.4 Hz, *J*_H,H_ = 1.0 Hz, 1CH_arom_), 7.50–7.51 (m, 2CH_arom_), 7.69–7.75 (m, 2CH_arom_), 8.09 (dd, *J*_H,H_ = 7.4 Hz, *J*_H,H_ = 1.3 Hz, 1CH_arom_), 8.14 (dd, *J*_H,H_ = 7.4 Hz, *J*_H,H_ = 1.2 Hz, 1CH_arom_) ppm; ^13^C NMR δ 40.6 (Ph-*C*H), 119.9, 125.3, 125.7, 126.6, 127.0, 127.6, 127.7, 128.4, 129.0, 133.4, 134.2 (12CH_arom_, C=*C*H), 125.9, 131.7, 132.1, 136.8, 140.3, 141.8, 143.9 (6C_arom_, *C*=CH), 180.6, 181.3 (2C=O) ppm; IR ν: 3085 (w), 3063 (w), 1652 (vs, 2C=O), 1590 (s), 1573 (s), 1490 (m), 1453 (m), 1347 (m), 1335 (m), 1289 (vs), 1230 (m), 1150 (m), 1111 (w), 1074 (m), 839 (m), 880 (m), 815 (m), 710 (s), 696 (s) cm^−1^; UV–vis (CH_2_Cl_2_) λ_max_/nm (lg ε): 269 (4.42), 473 (3.12); anal. calcd for C_23_H_14_O_2_S_2_ (386.49): C, 71.48; H, 3.65; S, 16.59; found: C, 71.48; H, 3.70; S, 16.58.

**11-Chloro-6-hydroxy-4-phenyl-2-(thiophen-2-yl)-4*H*-naphtho[2,3-*g*]thiochromene-5,12-dione (4k):** Yield: 285 mg (59%). Dark red crystals; mp 162 °C (dec.); ^1^H NMR δ 5.78 (d, *J*_H,H_ = 6.7 Hz, thiophen-2-yl-C*H*), 6.48 (d, *J*_H,H_ = 6.8 Hz, C=C*H*), 6.96 (dd, *J*_H,H_ = 4.9 Hz, *J*_H,H_ = 3.8 Hz, 1CH_arom_), 7.01–7.11 (m, 2CH_arom_), 7.21 (dd, *J*_H,H_ = 4.9 Hz, *J*_H,H_ = 1.0 Hz, 1CH_arom_), 7.35 (dd, *J*_H,H_ = 4.9 Hz, *J*_H,H_ = 1.0 Hz, 1CH_arom_), 7.40 (dd, *J*_H,H_ = 4.9 Hz, *J*_H,H_ = 1.0 Hz, 1CH_arom_), 7.76–7.80 (m, 1CH_arom_), 7.87–7.89 (m, 1CH_arom_), 8.59 (d, *J*_H,H_ = 8.2 Hz, 1CH_arom_), 8.63 (d, *J*_H,H_ = 8.2 Hz, 1CH_arom_), 14.98 (s, OH) ppm; ^13^C NMR δ 34.5 (thiophen-2-yl-*C*H), 118.7, 125.0, 125.5, 125.7, 125.8, 126.2, 127.2, 127.5, 127.8, 130.1, 132.2 (10CH_arom_, C=*C*H), 108.8, 122.5, 127.6, 128.5, 134.8, 135.2, 139.9, 143.5, 142.1, 147.4, 162.6 (10C_arom_, *C*=CH), 179.0, 184.0 (2C=O) ppm; IR ν: 3022 (w), 2953 (s), 2923 (w), 1655 (s, 2C=O), 1601 (s), 1584 (s), 1565 (s), 1491 (m), 1427 (s), 1397 (s), 1360 (s), 1360 (m), 1333 (m), 1242 (vs), 1170 (m), 902 (s), 852 (m), 832 (m), 758 (m), 699 (vs) cm^−1^; UV–vis (CH_2_Cl_2_) λ_max_/nm (lg ε): 245 (4.69), 294 (4.32), 487 (3.91); anal. calcd for C_27_H_15_ClO_3_S_2_ (486.99): C, 66.59; H, 3.10; S, 13.17; found: C, 66.39; H, 3.07; S, 13.06.

**(4a*SR*,10a*RS*)-10a-Methyl-2,4-diphenyl-4*H*-benzo[*g*]thiochromene-5,10-(4a*H*,10a*H*)-dione (6):** Yield: 145 mg (37%). Yellow-brown viscous oil; ^1^H NMR δ 1.94 (s, CH_3_), 3.76 (d, *J*_H,H_ = 4.8 Hz, 1CH), 4.18 (t, *J*_H,H_ = 4.5 Hz, 1CH), 6.50 (d, *J*_H,H_ = 4.3 Hz, 1CH), 7.08–7.12 (m, 1CH), 7.16–7.19 (m, 2CH), 7.30–7.38 (m, 5CH), 7.54–7.57 (m, 2CH), 7.58–7.61 (m, 2CH), 7.79–7.82 (m, 1CH), 7.91–7.93 (m, 1CH) ppm; ^13^C NMR δ 26.4 (CH_3_), 42.7 (CH-Ph), 55.9 (C_q_), 58.0 (CH-S), 120.0, 126.3, 126.8, 126.9, 127.3, 128.0, 128.5, 128.6, 129.5, 133.8, 134.2 (14CH_arom_, C=*C*H), 132.4, 134.4, 134.6, 138.9, 139.6 (4C_arom_, *C*=CH), 193.9, 194.2 (2C=O) ppm; IR ν: 3062 (w), 3034 (w), 2957 (w), 1696 (vs) and 1691 (s, 2C=O), 1614 (s), 1489 (m), 1451 (m), 1266 (s), 1013 (m), 979 (m), 761 (s), 692 (s) cm^–1^; ESIMS (for C_26_H_20_O_2_S): 395 (100, [M − 1]^−^).

## Supporting Information

CCDC-1838975 contains the supplementary crystallographic data for this paper. These data can be obtainded free of charge from the Cambridge Crystallographic Data Centre via http://www.ccdc.cam.ac.uk/structures.

File 1 Experimental data for selected compounds **4**, details of the crystal structure determination, and copies of ^1^H and ^13^C NMR spectra for all products.
