# Supplementary material for: First thia-Diels–Alder reactions of thiochalcones with 1,4-quinones
Source: Beilstein J Org Chem. 2018 Jul 19;14:1834–9. doi: 10.3762/bjoc.14.156 (PMC6071716; doi:10.3762/bjoc.14.156)
Supplement: File 1 — Experimental data for selected compounds 4, details of the crystal structure determination, and copies of 1H and 13C NMR spectra for all products. [file Beilstein_J_Org_Chem-14-1834-s001.pdf]

Supporting Information
for
First thia-Diels–Alder reactions of thiochalcones with 1,4-quinones

Grzegorz Mlostoń*[1], Katarzyna Urbaniak[1], Paweł Urbaniak[2], Anna Marko[1], Anthony Linden[3], and Heinz Heimgartner[3]

Address: [1]Department of Organic and Applied Chemistry, University of Łódź, Tamka 12, PL 91-403 Łódź, Poland, [2]Department of Inorganic and Analytical Chemistry, University of Łódź, Tamka 12, PL 91-403 Łódź, Poland and [3]Department of Chemistry, University of Zurich, Winterthurerstrasse 190, CH-8057 Zurich, Switzerland

Email: Grzegorz Mlostoń - grzegorz.mloston@chemia.uni.lodz.pl

*Corresponding author

Experimental data for selected compounds 4, details of the crystal structure determination, and copies of ^{1}H and ^{13}C NMR spectra for all products.

Experimental data for compounds 4d–j, 4l–n, details of the crystal structure determination, and the original ^{1}H and ^{13}C NMR spectra for all described compounds

2,4-Bis(thiophen-2-yl)-4*H*-benzo[*g*]thiochromene-5,10-dione (**4d**): Yield: 370 mg (94%). Red-orange crystals; mp = 158 °C (dec.); ^{1}H NMR: δ 5.76 (*d*, $J_{H,H}$ = 6.7 Hz, thiophen-2-yl-C*H*); 6.48 (*d*, $J_{H,H}$ = 6.5 Hz, C=C*H*); 6.94 (*dd*, $J_{H,H}$ = 5.0 Hz, $J_{H,H}$ = 3.5 Hz, 1CH_{arom}); 7.05 (*d*, $J_{H,H}$ = 3.5 Hz, 1CH_{arom}); 7.09 (*dd*, $J_{H,H}$ = 5.2 Hz, $J_{H,H}$ = 3.7 Hz, 1CH_{arom}); 7.19 (*dd*, $J_{H,H}$ = 5.0 Hz, $J_{H,H}$ = 1.1 Hz, 1CH_{arom}); 7.34 (*dd*, $J_{H,H}$ = 5.0 Hz, $J_{H,H}$ = 1.1 Hz, 1CH_{arom}); 7.39 (*dd*, $J_{H,H}$ = 3.7 Hz, $J_{H,H}$ = 1.1 Hz, 1CH_{arom}); 7.72–7.79 (*m*, 2CH_{arom}); 8.14 (*dd*, $J_{H,H}$ = 7.5 Hz, $J_{H,H}$ = 1.1 Hz, 1CH_{arom}); 8.18 (*dd*, $J_{H,H}$ = 7.4 Hz, $J_{H,H}$ = 1.2 Hz, 1CH_{arom}) ppm; ^{13}C NMR: δ 34.9 (thiophen-2-yl-*C*H); 118.8, 125.4, 125.7, 126.1, 126.7, 127.1, 127.2, 127.7, 133.5, 134.4 (10CH_{arom}, C=*C*H); 127.3, 131.8 132.0, 134.4, 135.8, 139.9, 143.6 (6C_{arom}, *C*=CH); 180.4, 181.2 (2C=O) ppm; IR: ν 3069*w*, 3009*w*, 1652*vs* (2C=O), 1611*m*, 1589*s*, 1567*m*, 1424*m*, 1338*m*, 1327*m*, 1281*vs*, 1228*m*, 1148*m*, 1108*m*, 1047*m*, 854*m*, 820*s*, 815*m*, 708*s* cm^{-1}; UV-VIS (CH_2Cl_2): λ_{max}/nm (lg ε) 252 (4.48); 459 (3.29); anal. calcd for $C_{21}H_{12}O_2S_3$ (392.51): C, 64.26; H, 3.08; S, 24.51; found: C, 64.40; H, 3.19; S, 24.53.

2,4-Diphenyl-4*H*-naphtho[2,3-*g*]thiochromene-5,12-dione (**4e**): Yield: 415 mg (96%). Red-orange crystals; mp = 222 °C (dec.); ^{1}H NMR: δ 5.55 (*d*, $J_{H,H}$ = 6.4 Hz, Ph-C*H*); 6.38 (*d*, $J_{H,H}$ = 6.4 Hz, C=C*H*); 7.22–7.24 (*m*, 1CH_{arom}); 7.33–7.34 (*m*, 2CH_{arom}); 7.41–7.43 (*m*, 3CH_{arom}); 7.55–7.57 (*m*, 2CH_{arom}); 7.63–7.65 (*m*, 2CH_{arom}); 7.68–7.70 (*m*, 2CH_{arom}); 8.08–8.10 (*m*, 2CH_{arom}); 8.63 (*s*, 1CH_{arom}); 8.68 (*s*, 1CH_{arom}) ppm; ^{13}C NMR: δ 41.1 (Ph-*C*H); 121.1, 126.7, 127.5, 128.4, 128.8, 128.9, 129.0, 129.2, 129.3, 129.5, 129.7, 130.1, 130.2 (16CH_{arom}, C=*C*H); 128.1, 128,4, 132.2, 134.6, 135.2, 137.4, 138.0, 142.3, 146.0 (8C_{arom}, *C*=CH); 180.4, 181.0 (2C=O) ppm; IR: ν 3085*w*, 3063*w*, 1660*vs* (2C=O), 1615*s*, 1582*s*, 1491*m*, 1445*m*, 1401*m*, 1355*m*, 1283*vs*, 1255*m*, 1197*m*, 1074*w*, 854*m*, 754*s*, 695*s* cm^{-1}; UV-VIS (CH_2Cl_2): λ_{max}/nm (lg ε) 304 (4.45), 420 (3.81); anal. calcd for $C_{29}H_{18}O_2S$ (430.52): C, 80.91; H, 4.21; S, 7.45; found: C, 80.96; H, 4.32; S, 7.23.

2-Phenyl-4-(thiophen-2-yl)-4*H*-naphto[2,3-*g*]thiochromene-5,12-dione (**4f**): Yield: 390 mg (89%). Red-orange crystals; mp = 215 °C (dec.); ^{1}H NMR: δ 5.88 (*d*, $J_{H,H}$ = 6.6 Hz, thiophen-2-yl-C*H*); 6.41 (*d*, $J_{H,H}$ = 6.6 Hz, C=C*H*); 6.95–6.97 (*m*, 1CH_{arom}); 7.10 (*d*, $J_{H,H}$ = 3.4 Hz, 1CH_{arom}); 7.20 (*d*, $J_{H,H}$ = 5.1 Hz, 1CH_{arom}); 7.43–7.45 (*m*, 3CH_{arom}); 7.66–7.70 (*m*, 4CH_{arom}); 8.00–8.01 (*m*, 2CH_{arom}); 8.66 (*s*, 1CH_{arom}); 8.69 (*s*, 1CH_{arom}) ppm. ^{13}C NMR: δ 35.4 (thiophen-2-yl-*C*H); 120.0, 125.3, 125.6, 127.0, 127.1, 128.8, 129.2, 129.3, 129.5, 129.6, 129.8, 130.1, 130.2 (14CH_{arom}, C=*C*H); 128.0, 128.3, 133.7, 134.6, 135.2, 136.9, 137.2, 144.2, 145.7 (8C_{arom}, *C*=CH); 180.2, 181.3 (2C=O) ppm. IR: ν 3060*w*, 3028*w*, 1659*vs* (2C=O), 1615*s*, 1582*s*, 1492*m*, 1459*s*, 1403*m*, 1351*m*, 1283*vs*, 1255*m*, 1197*m*, 1103*w*, 1078*w*, 913*m*, 853*s*, 762*s*, 747*s*, 712*s*, 695m cm^{-1}; UV-VIS (CH_2Cl_2): λ_{max}/nm (lg ε) 275 (4.33), 304 (4.46), 421 (3.48); anal. calcd for $C_{27}H_{16}O_2S_2$ (436.54): C, 74.29; H, 3.69; S, 14.69; found: C, 74.06; H, 3.90; S, 14.55.

4-Phenyl-2-(thiophen-2-yl)-4*H*-naphtho[2,3-g]thiochromene-5,12-dione (**4g**): Yield: 385 mg (88%). Red-orange crystals; mp = 226 °C (dec.); ^{1}H NMR: δ 5.53 (*d*, $J_{H,H}$ = 6.5 Hz, Ph-C*H*); 6.45 (*d*, $J_{H,H}$ = 6.5 Hz, C=C*H*); 7.08–7.11 (*m*, 2CH_{arom}); 7.23–7.24 (*m*, 1CH_{arom}); 7.30–7.34 (*m*, 2CH_{arom}); 7.38 (*dd*, $J_{H,H}$ = 3.7 Hz, $J_{H,H}$ = 1.0 Hz, 1CH_{arom}); 7.53–7.55 (*m*, 2CH_{arom}); 7.68–7.71 (*m*, 2CH_{arom}); 8.08–8.10 (*m*, 2CH_{arom}); 8.64 (*s*, 1CH_{arom}); 8.67 (*s*, 1CH_{arom}) ppm. ^{13}C NMR: δ 40.7 (Ph-*C*H); 120.0, 125.3, 125.7, 127.6, 127.7, 128.5, 128.9, 129.2, 129.5, 129.6, 129.8, 130.1, 130.2 (14CH_{arom}, C=C*H*); 126.0, 128.0, 128.3, 134.6, 135.2, 138.5, 140.3, 141.8, 145.5 (8C_{arom}, *C*=CH); 180.3, 181.0 (2C=O) ppm. IR: ν 3069*w*, 3018*w*, 1661*vs* and 1644*s* (2C=O), 1616*s*, 1587*s*, 1581*s*, 1459*s*, 1404*m*, 1360*m*, 1288*vs*, 1255*m*, 1232*m*, 1223*m*, 1199*m*, 1042*m*, 872*m*, 846*m*, 754*s*, 716*s*, 695*m* cm^{-1}; UV-VIS (CH_2Cl_2): λ_{max}/nm (lg ε) 301 (4.68), 412 (3.82); anal. calcd for $C_{27}H_{16}O_2S_2$ (436.54): C, 74.29; H, 3.69; S, 14.69; found: C, 74.26; H, 3.71; S, 14.52.

2,4-Bis(thiophen-2-yl)-4*H*-naphtho[2,3-*g*]thiochromene-5,12-dione (**4h**): Yield: 420 mg (95%). Red-orange crystals; mp = 205 °C (dec.); ^{1}H NMR: δ 5.84 (*d*, $J_{H,H}$ = 6.7 Hz, thiophen-2-yl-C*H*); 6.51 (*d*, $J_{H,H}$ = 6.7 Hz, C=C*H*); 6.95 (*dd*, $J_{H,H}$ = 5.1 Hz, $J_{H,H}$ = 3.7 Hz, 1CH_{arom}); 7.08–7.12 (*m*, 2CH_{arom}); 7.19 (*dd*, $J_{H,H}$ = 5.1 Hz, $J_{H,H}$ = 1.0 Hz, 1CH_{arom}); 7.35 (*dd*, $J_{H,H}$ = 5.1 Hz, $J_{H,H}$ = 1.0 Hz, 1CH_{arom}); 7.42 (*dd*, $J_{H,H}$ = 3.7 Hz, $J_{H,H}$

= 1.0 Hz, $1CH_{arom}$); 7.70–7.73 (*m*, $2CH_{arom}$); 8.06–8.08 (*m*, $2CH_{arom}$); 8.68 (*s*, $1CH_{arom}$); 8.70 (*s*, $1CH_{arom}$) ppm. ^{13}C NMR: δ 35.1 (thiophen-2-yl-*C*H); 118.9, 125.4, 125.7, 125.8, 126.1, 127.1, 127.8, 129.3, 129.6, 129.7, 129.8, 130.2, 130.3 ($12CH_{arom}$, C=*C*H); 127.4, 128.0, 128.2, 134.6, 135.2, 137.5, 140.0, 143.6, 145.2 ($8C_{arom}$, *C*=CH); 180.3, 180.9 (2C=O) ppm. IR: ν 3063*w*, 3028*w*, 1662*vs* and 1645*s* (2C=O), 1615*s*, 1583*s*, 1459*s*, 1403*m*, 1348*m*, 1286*vs*, 1255*m*, 1198*m*, 848*m*, 824*m*, 808*m*, 760*s*, 749*m*, 690*m* cm^{-1}; UV-VIS (CH_2Cl_2): λ_{max}/nm (lg ε) 302 (4.83), 420 (3.89), 773 (2.29); anal. calcd for $C_{25}H_{14}O_2S_3$ (442.58): C, 67.85; H, 3.19; S, 21.74; found: C, 67.55; H, 3.29; S, 21.68.

11-Chloro-6-hydroxy-2,4-diphenyl-4*H*-naphtho[2,3-*g*]thiochromene-5,12-dione (**4i**): Yield: 310 mg (65%). Dark red crystals; mp = 164 °C (dec.); ^{1}H NMR: δ 5.47 (*d*, $J_{H,H}$ = 6.4 Hz, Ph-C*H*); 6.34 (*d*, $J_{H,H}$ = 6.4 Hz, C=C*H*); 7.24–7.25 (*m*, $2CH_{arom}$); 7.33–7.36 (*m*, $2CH_{arom}$); 7.40–7.44 (*m*, $2CH_{arom}$); 7.54–7.56 (*m*, $2CH_{arom}$); 7.60–7.62 (*m*, $2CH_{arom}$); 7.73–7.75 (*m*, $1CH_{arom}$); 7.82–7.84 (*m*, $1CH_{arom}$); 8.15 (*d*, $J_{H,H}$ = 8.3 Hz, $1CH_{arom}$); 8.60 (*d*, $J_{H,H}$ = 8.3 Hz, $1CH_{arom}$); 14.94 (*s*, OH) ppm. ^{13}C NMR: δ 40.6 (Ph-*C*H); 121.0, 124.9, 126.7, 127.5, 127.6, 128.3, 128.8, 129.0, 129.1, 130.0, 132.0 ($14CH_{arom}$, C=*C*H); 108.8, 128.4, 128.6, 132.1, 132.3, 134.8, 135.7, 137.3, 142.2, 148.4, 162.4 ($10C_{arom}$, *C*=CH); 179.2, 184.4 (2C=O) ppm. IR: ν 3056*w*, 3025*w*, 1665*vs* and 1640*s* (2C=O), 1599*s*, 1576*vs*, 1490*s*, 1444*s*, 1396*s*, 1359*s*, 1335*s*, 1241*vs*, 1193*m*, 1169*m*, 898*s*, 871*s*, 857*s*, 758*s*, 743*s*, 698*s* cm^{-1}; UV-VIS (CH_2Cl_2): λ_{max}/nm (lg ε) 245 (4.81), 280 (4.22), 313 (4.23), 484 (3.98); anal. calcd for $C_{29}H_{17}ClO_3S$ (480.96): C, 72.42; H, 3.56; S, 6.67; found: C, 72.19; H, 3.76; S, 6.58.

11-Chloro-6-hydroxy-2-phenyl-4-(thiophen-2-yl)-4*H*-naphtho[2,3-*g*]thiochromene-5,12-dione (**4j**): Yield: 290 mg (60%). Dark red crystals; mp = 162 °C (dec.); ^{1}H NMR: δ 5.60 (*d*, $J_{H,H}$ = 6.5 Hz, thiophen-2-yl-C*H*); 5.95 (*d*, $J_{H,H}$ = 6.5 Hz, C=C*H*); 6.71 (*dd*, $J_{H,H}$ = 5.1 Hz, $J_{H,H}$ = 3.7 Hz, $1CH_{arom}$); 6.81 (*dd*, $J_{H,H}$ = 3.7 Hz, $J_{H,H}$ = 1.0 Hz, $1CH_{arom}$); 7.07–7.11 (*m*, $4CH_{arom}$); 7.16–7.18 (*m*, $2CH_{arom}$); 7.44–7.46 (*m*, $2CH_{arom}$); 8.32 (*d*, $J_{H,H}$ = 8.3 Hz, $1CH_{arom}$); 8.41 (*d*, $J_{H,H}$ = 8.3 Hz, $1CH_{arom}$); 14.94 (*s*, OH) ppm. ^{13}C NMR: δ 34.8 (thiophen-2-yl-*C*H); 119.8, 124.5, 125.2, 125.8, 126.8, 127.0, 127.1, 128.7, 128.9, 129.2 ($12CH_{arom}$, C=*C*H); 109.1, 122.8, 128.4, 128.5, 133.6, 134.1, 134.7, 137.3, 144.4, 147.7, 162.1 ($10C_{arom}$, *C*=CH); 178.1, 184.1

(2C=O) ppm. IR: ν 3050*w*, 3025*w*, 1653*vs* (2C=O), 1622*m*, 1597*vs*, 1571*vs*, 1490*s*, 1427*s*, 1394*s*, 1362*s*, 1332*s*, 1243*vs*, 1225*s*, 1192*m*, 1112*m*, 957*m*, 902*s*, 871*m*, 859*m*, 761*s*, 709*s*, 692*s* cm^{-1}; UV-VIS (CH_2Cl_2): λ_{max}/nm (lg ε) 246 (4.87), 312 (4.28), 492 (4.07), 981 (2.30); anal. calcd for $C_{27}H_{15}ClO_3S_2$ (486.99): C, 66.59; H, 3.10; S, 13.17; found C, 66.62; H, 3.13; S, 13.10.

11-Chloro-6-hydroxy-2,4-bis(thiophen-2-yl)-4*H*-naphtho[2,3-*g*]thiochromene-5,12-dione (**4l**): Yield: 285 mg (58%). Dark red crystals; mp = 168 °C (dec.); ^{1}H NMR: δ 5.45 (*d*, $J_{H,H}$ = 6.5 Hz, Ph-C*H*); 6.43 (*d*, $J_{H,H}$ = 6.5 Hz, C=C*H*); 7.08 (*dd*, $J_{H,H}$ = 5.0 Hz, $J_{H,H}$ = 3.7 Hz, 1CH_{arom}); 7.23–7.38 (*m*, 3CH_{arom}); 7.52–7.53 (*m*, 2CH_{arom}); 7.67–7.70 (*m*, 2CH_{arom}); 7.86–7.88 (*m*, 2CH_{arom}); 8.55 (*d*, $J_{H,H}$ = 8.2 Hz, 1CH_{arom}); 8.64 (*d*, $J_{H,H}$ = 8.2 Hz, 1CH_{arom}); 14.93 (*s*, OH) ppm. ^{13}C NMR: δ 40.3 (Ph-*C*H); 119.9, 124.5, 125.2, 125.4, 127.0, 127.5, 127.6, 128.6, 128.9, 129.2 131.4 (12CH_{arom}, C=*C*H); 108.9, 122.7, 126.2, 127.2, 128.5, 134.7, 135.6, 140.4, 142.1, 147.5, 162.1 (10C_{arom}, *C*=CH); 178.0, 184.2 (2C=O) ppm. IR: ν 3088*w*, 2924*s*, 2854*s*, 1655*vs*, (2C=O), 1575*vs*, 1489*s*, 1425*s*, 1396*s*, 1380*s*, 1357*s*, 1336*m*, 1241*s*, 1168*m*, 902*s*, 902*s*, 863*m*, 809*m*, 765*s*, 742*s*, 699*vs*, 680*m* cm^{-1}; UV-VIS (CH_2Cl_2): λ_{max}/nm (lg ε) 245 (4.79), 294 (4.44), 490 (4.05); anal. calcd for $C_{25}H_{13}ClO_3S_3$ (493.01): C 60.90, H 2.66, S 19.51; found: C 60.79, H 2.79, S 19.77.

2,4-Diphenyl-4*H*-thiochromene-5,8-dione (**4m**): Yield: 230 mg (70%). Maroon colored, viscous oil; ^{1}H NMR: δ 5.37 (*d*, $J_{H,H}$ = 7.0 Hz, Ph-C*H*); 6.44 (*d*, $J_{H,H}$ = 7.0 Hz, C=C*H*); 6.61, 6.72 (*AB-system*, $J_{H,H}$ = 8.5 Hz, 2CH_{arom}); 7.23–7.24 (*m*, 1CH_{arom}); 7.29–7.46 (*m*, 7CH_{arom}); 7.58–7.60 (*m*, 2CH_{arom}) ppm. ^{13}C NMR: δ 40.5 (Ph-*C*H); 120.9, 126.7, 127.6, 128.3, 128.8, 128.9, 129.1, 135.6, 137.5 (12CH_{arom}, C=*C*H); 131.9, 134.2, 136.3, 142.0, 142.1 (4C_{arom}, *C*=CH); 189.9, 183.4 (2C=O) ppm. IR: ν 3053*m*, 3025*m*, 2923*m*, 1645*vs* (C=O), 1569*s*, 1490*s*, 1444*m*, 1289*s*, 1119*m*, 1074*m*, 1033*m*, 929*m*, 903*m*, 756*s*, 695*s* cm^{-1}; UV-VIS (CH_2Cl_2): λ_{max}/nm (lg ε) 233 (4.37), 359 (3.43), 499 (2.99); ESI-MS (for $C_{21}H_{14}O_2S$): 352 (100, $[M–1+Na]^+$), 353 (27, $[M+Na]^+$).

2-Phenyl-4-(thiophen-2-yl)-4*H*-thiochromene-5,8-dione (**4n**): Yield: 215 mg (64%). Maroon colored, viscous oil; ^{1}H NMR: δ 5.56 (*d*, $J_{H,H}$ = 6.7 Hz, thiophen-2-yl-C*H*);

6.34 (*d*, $J_{H,H}$ = 6.7 Hz, C=C*H*); 6.82, 6.85 (*AB-system*, $J_{H,H}$ = 10.0 Hz, $2CH_{arom}$); 6.99 (*dd*, $J_{H,H}$ = 5.0 Hz, $J_{H,H}$ = 3.5 Hz, $1CH_{arom}$); 7.00–7.02 (*m*, $1CH_{arom}$); 7.21 (*dd*, $J_{H,H}$ = 5.0 Hz, $J_{H,H}$ = 1.1 Hz, $1CH_{arom}$); 7.42–7.44 (*m*, $3CH_{arom}$); 7.60–7.62 (*m*, $2CH_{arom}$) ppm. ^{13}C NMR: δ 34.8 (thiophen-2-yl-*C*H); 119.9, 125.4, 125.5, 127.0, 127.2, 128.9, 129.3, 135.8, 137.3 ($10CH_{arom}$, C=*C*H); 133.1, 133.4, 137.5, 41.7, 144.2 ($4C_{arom}$, *C*=CH); 182.7, 183.3 (2C=O) ppm. IR: ν 3098*w*, 3073*w*, 3025*m*, 1666*s* (C=O), 1645*vs* (C=O), 1574*s*, 1489*s*, 1442*m*, 1366*m*, 1292*s*, 1220*m*, 1119*m*, 1030*m*, 926*m*, 903*m*, 830*s*, 761*s*, 691*s* cm^{-1}; UV-VIS (CH_2Cl_2): λ_{max}/nm (lg ε) 237 (4.46), 355 (3.45), 405 (3.37), 486 (3.18); ESI-MS (for $C_{19}H_{12}O_2S_2$): 335 (100, $[M–1]^+$); 336 (25, $[M]^+$).

X-ray crystallography

Crystal data for **4k**: $C_{27}H_{15}ClO_3S_2$, M_r = 486.96, red, prism, 0.06 × 0.15 × 0.27 mm, T = 160(1) K, triclinic, $P\bar{1}$, Z = 2, a = 7.3545(3), b = 9.6157(3), c = 16.2246(6) Å, α = 80.036(3), β = 84.669(3), γ = 68.352(3), V = 1049.84(7) $Å^3$, D_x = 1.540 g cm^{-3}, μ(MoKα) = 0.411 mm^{-1}, ω scans, $2\theta_{(max)}$ = 60.7°, transmission factors (min; max) = 0.520; 1.000, 25895 reflections measured, 5808 symmetry independent reflections, 5095 reflections with $I > 2\sigma(I)$, 5808 reflections used in refinement, 312 parameters refined, 8 restraints, $R(F)$ [$I > 2\sigma(I)$ reflections] = 0.0332, $wR(F^2)$ (all data) = 0.0923, $w = [\sigma^2(F_o^2) + (0.0457P)^2 + 0.3507P]^{-1}$ where $P = (F_o^2 + 2F_c^2)/3$, goodness of fit = 1.057, final Δ_{max}/σ = 0.001, $\Delta\rho$(max; min) = 0.41; –0.25 e $Å^{-3}$. Crystals from petroleum ether/CH_2Cl_2.

All measurements were made on a Rigaku Oxford Diffraction SuperNova area-detector diffractometer [S1], using MoKα radiation (λ = 0.71073 Å) from a microfocus X-ray source and an Oxford Instruments Cryojet XL cooler. Data reduction was performed with CrysAlisPro [S1]. The intensities were corrected for Lorentz and polarization effects, and a numerical absorption correction [S2] was applied. Equivalent reflections were merged. The data collection and refinement parameters are given above, and views of the two conformations of molecule **4k** are shown in Figure 1 and in Figure S1. The structure was solved by dual space methods using SHELXT-2018 [S3], which revealed the positions of all non-H-atoms. The S-atom of the thiophene ring is disordered as a result of slight but opposite directions of envelope puckering of the ring. Two positions were defined for the S-atom and the

site occupation factor of the major conformation refined to 0.78(2). Similarity restraints were applied to the chemically equivalent C–S bond lengths, while the atomic displacement parameters of these two S-atoms were restraint to be similar. The hydroxy H-atom was placed in the position indicated by a difference electron density map and its position was allowed to refine together with an isotropic displacement parameter. All remaining H-atoms were placed in geometrically calculated positions and refined by using a riding model where each H-atom was assigned a fixed isotropic displacement parameter with a value equal to $1.2U_{eq}$ of its parent C-atom. The refinement of the structure was carried out on F^2 by using full-matrix least-squares procedures, which minimized the function $\Sigma w(F_o^2 - F_c^2)^2$. The weighting scheme was based on counting statistics and included a factor to downweight the intense reflections. A correction for secondary extinction was not applied. Neutral atom scattering factors for non-H atoms were taken from Maslen, Fox and O'Keefe [S4], and the scattering factors for H-atoms were taken from Stewart, Davidson and Simpson [S5]. Anomalous dispersion effects were included in F_c [S6]; the values for f' and f'' were those of Creagh and McAuley [S7]. The values of the mass attenuation coefficients are those of Creagh and Hubbel [S8]. The *SHELXL-2018* program [S9] was used for all calculations. CCDC-1838975 contains the supplementary crystallographic data for this paper. These data can be obtained free of charge from The *Cambridge Crystallographic Data Centre*, via www.ccdc.cam.ac.uk/structures.

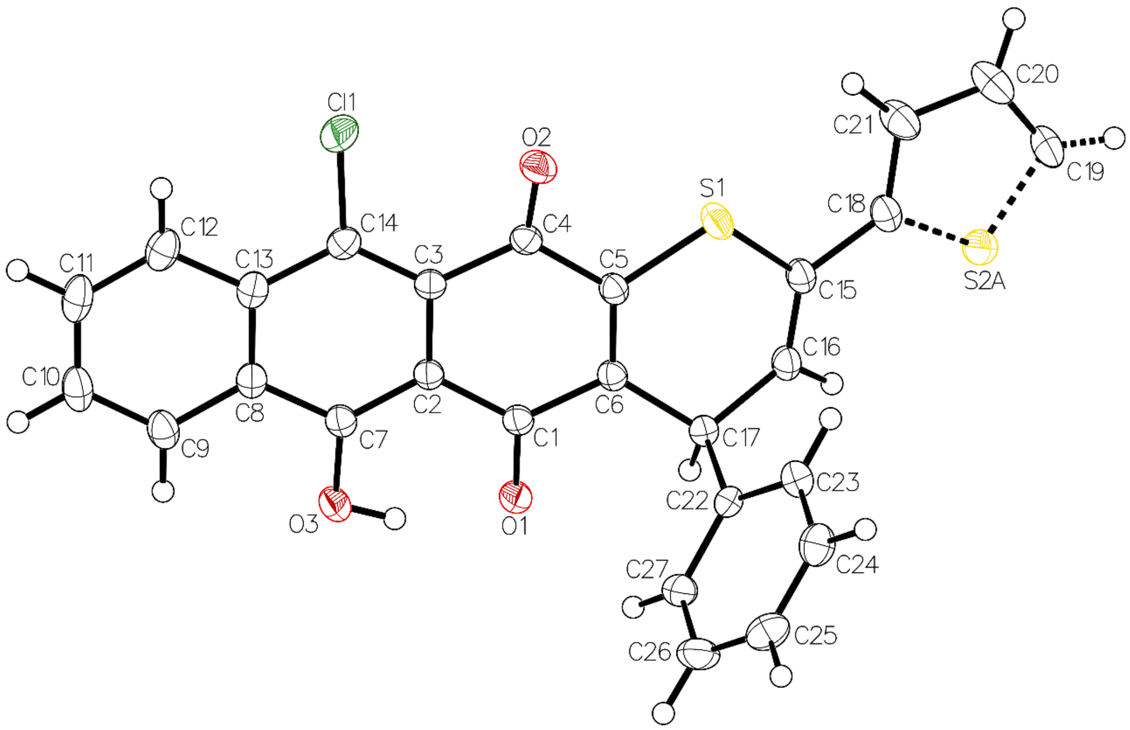

Figure S1: ORTEP plot [S11] of the molecular structure of **4k** showing the minor conformation of the disordered thiophene ring (50% probability ellipsoids; arbitrary numbering of the atoms).

References

[S1] CrysAlisPro, Version 1.171.39.46, Rigaku Oxford Diffraction, Chalgrove, Oxfordshire, England, 2018.

[S2] Coppens, P.; Leiserowitz, L.; Rabinovich, D. *Acta Crystallogr.* **1965**, *18*, 1035–1038.

[S3] Sheldrick, G. M. *Acta Crystallogr. Sect. A*, **2015**, *71*, 3–8.

[S4] Maslen, E. N.; Fox, A. G.; O'Keefe, M. A. in 'International Tables for Crystallography', Ed. Wilson, A. J. C. Kluwer Academic Publishers, Dordrecht, 1992, Vol. C, Table 6.1.1.1, pp. 477–486.

[S5] Stewart, R. F.; Davidson, E. R.; Simpson, W. T. *J. Chem. Phys.* **1965**, *42*, 3175–3187. doi: 10.1063/1.1696397.

[S6] Ibers, J. A.; Hamilton, W. C. *Acta Crystallogr.* **1964**, *17*, 781–782. doi: 10.1107/S0365110X64002067.

[S7] Creagh, D. C.; McAuley, W. J. in 'International Tables for Crystallography', Ed. Wilson, A. J. C. Kluwer Academic Publishers, Dordrecht, 1992, Vol. C, Table 4.2.6.8, pp. 219–222.

[S8] Creagh, D. C.; Hubbell, J. H. in 'International Tables for Crystallography', Ed. Wilson, A. J. C. Kluwer Academic Publishers, Dordrecht, 1992, Vol. C, Table 4.2.4.3, pp. 200–206.

[S9] Sheldrick, G. M. Acta Crystallogr. Sect. C, **2015**, *71*, 3–8. doi: 10.1107/S2053229614024218.

[S11] Johnson, C. K. ORTEP II, Report ORNL-5138, Oak Ridge National Laboratory, Oak Ridge, Tennessee, 1976.

Copies of the ^{1}H, ^{13}C NMR and UV–vis spectra for compounds 4a–m

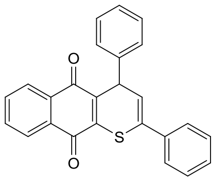

4a

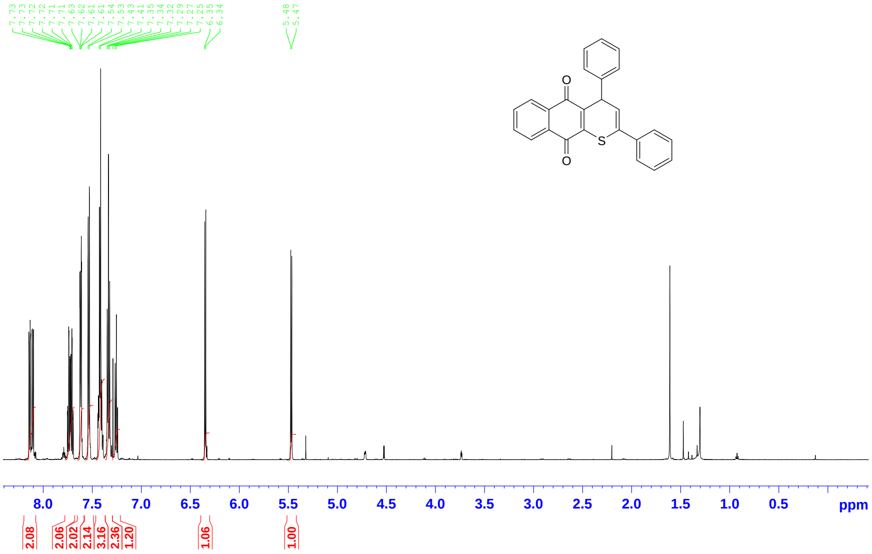

Figure S2: The ^{1}H NMR spectrum for 4a

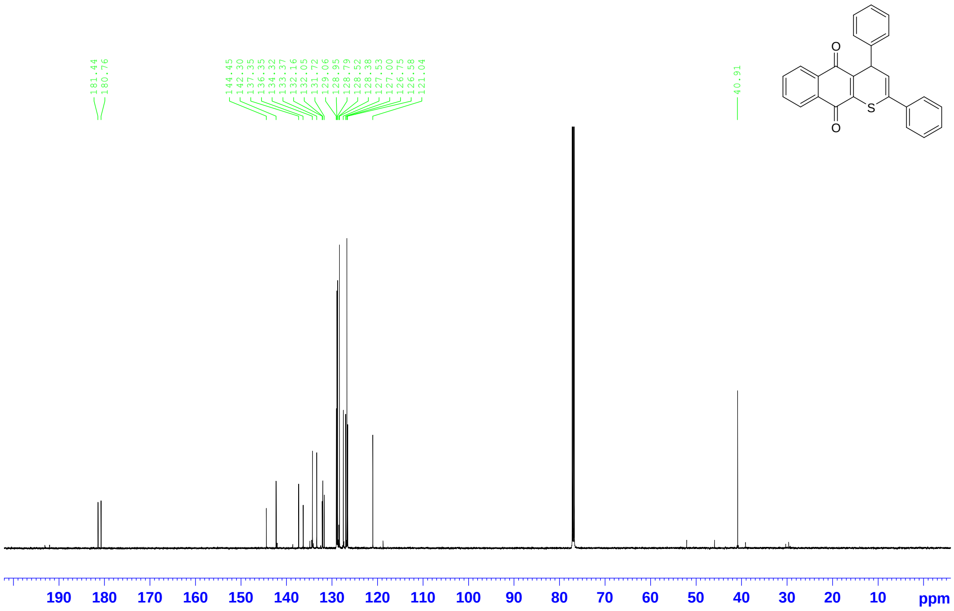

Figure S3: The ^{13}C NMR spectrum for 4a

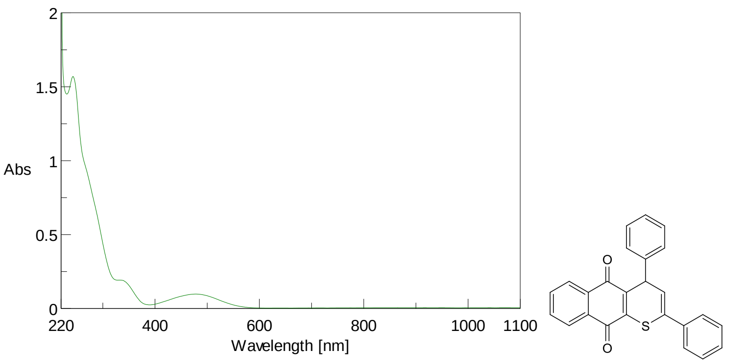

Figure S4: The UV-VIS spectrum for 4a

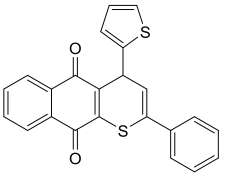

4b

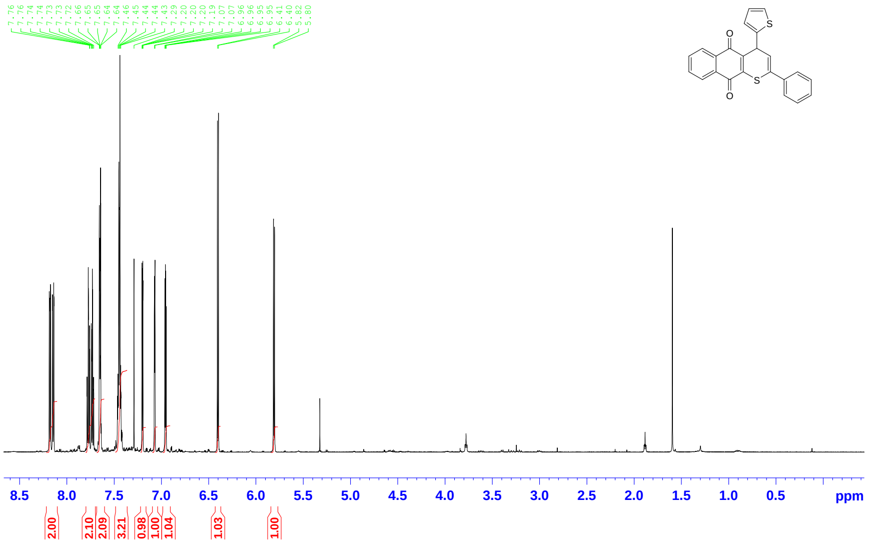

Figure S5: The ^{1}H NMR spectrum for 4b

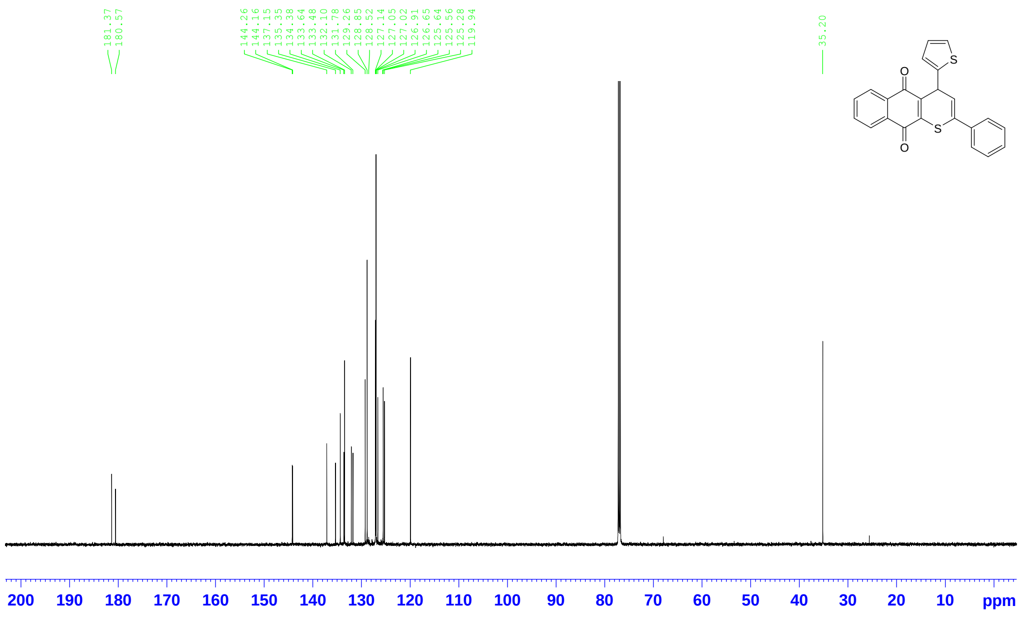

Figure S6: The ^{13}C NMR spectrum for 4b

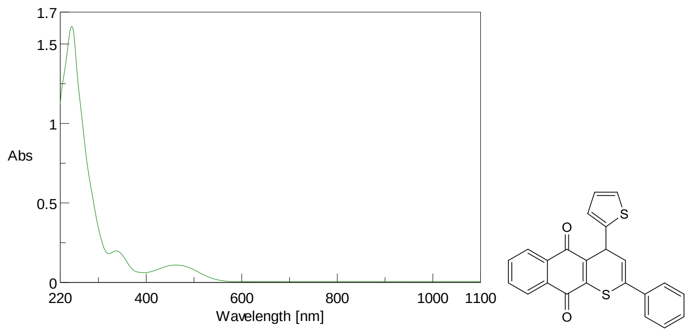

Figure S7: The UV-VIS spectrum for 4b

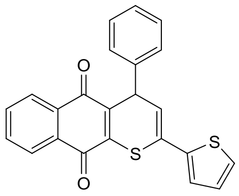

4c

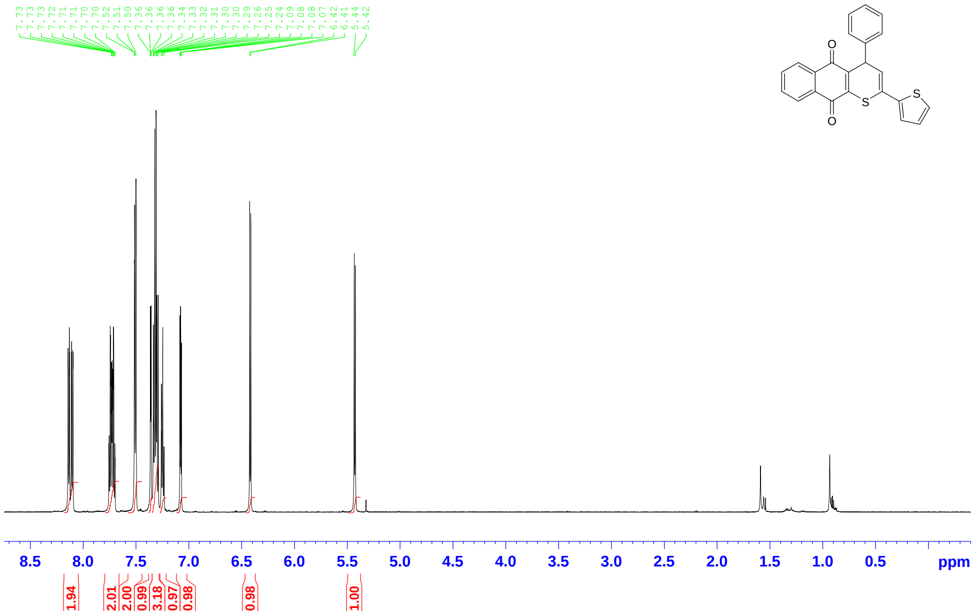

Figure S8: The ^{1}H NMR spectrum for 4c

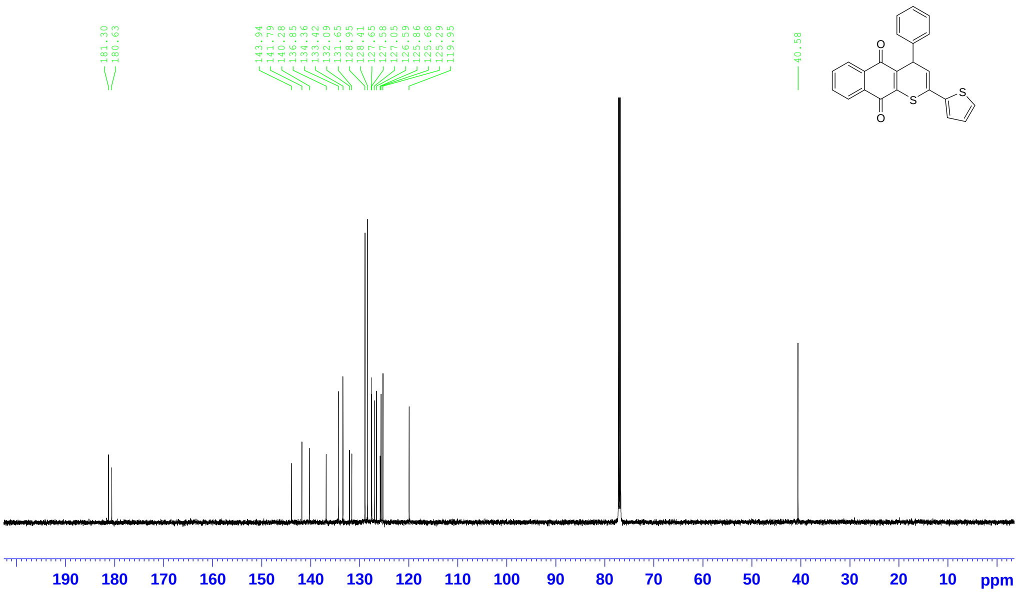

Figure S9: The ^{13}C NMR spectrum for 4c

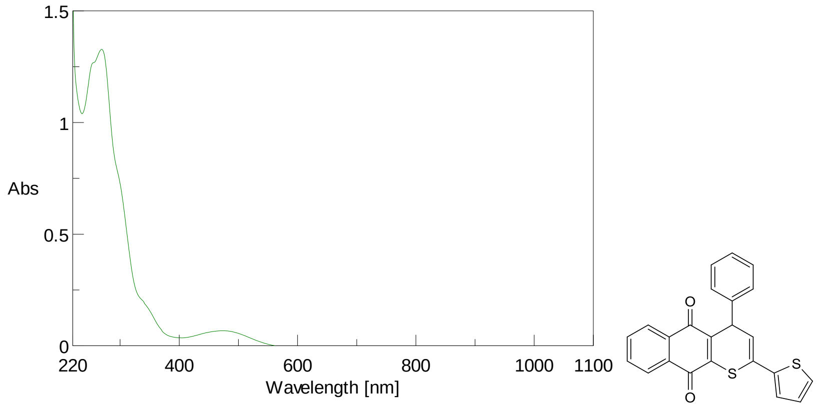

Figure S10: The UV-VIS spectrum for 4c

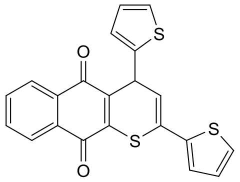

4d

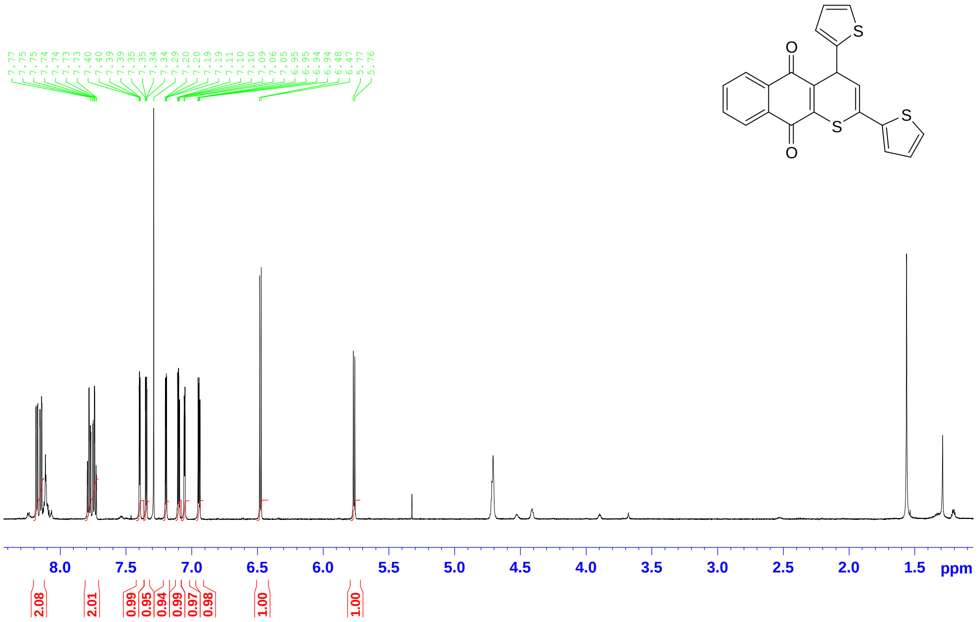

Figure S11: The ¹H NMR spectrum for 4d

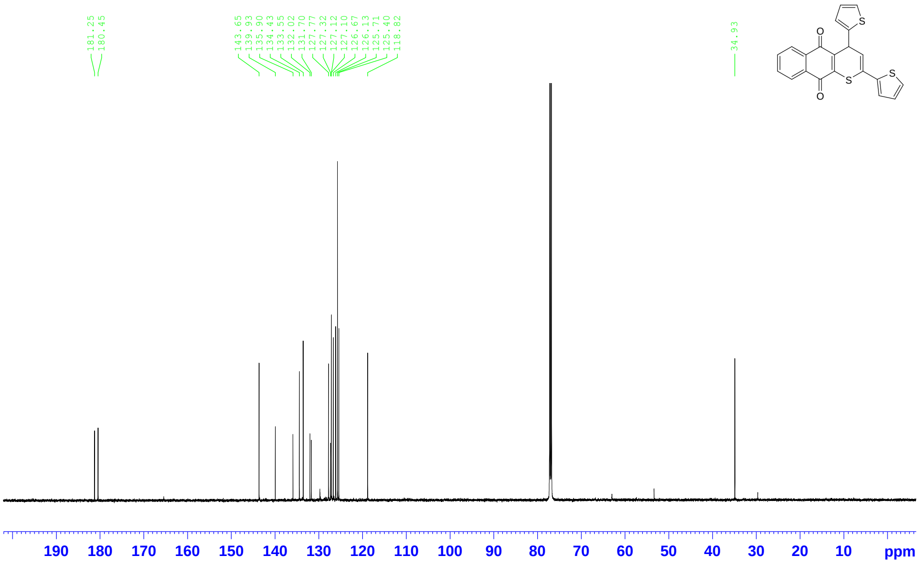

Figure S12: The ^{13}C NMR spectrum for 4d

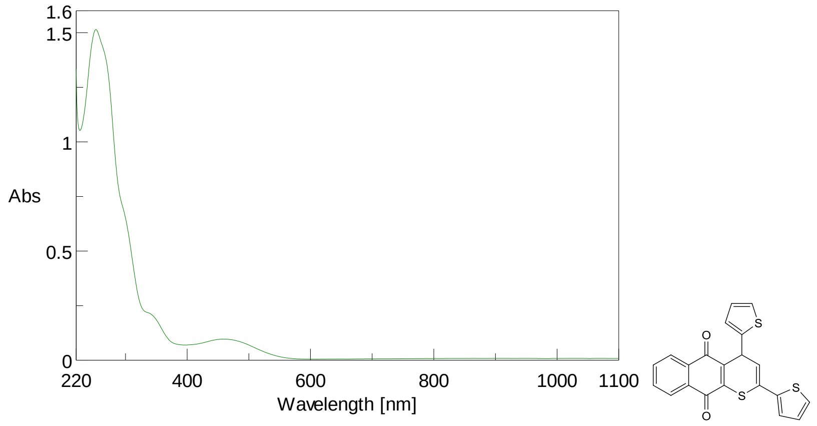

Figure S13: The UV-VIS spectrum for 4d

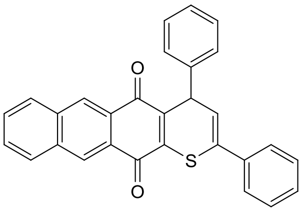

4e

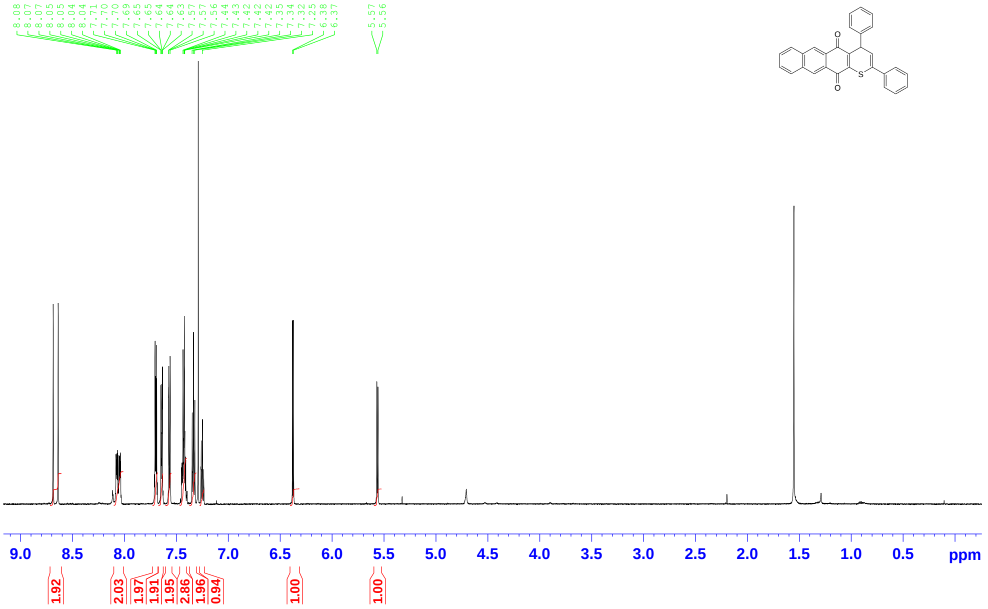

Figure S14: The ^{1}H NMR spectrum for 4e

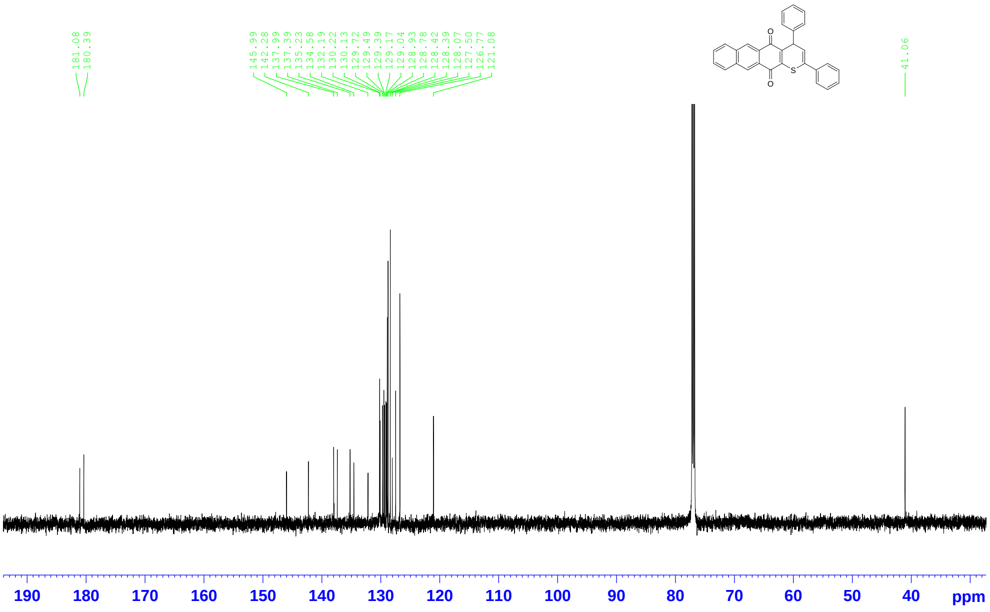

Figure S15: The ^{13}C NMR spectrum for 4e

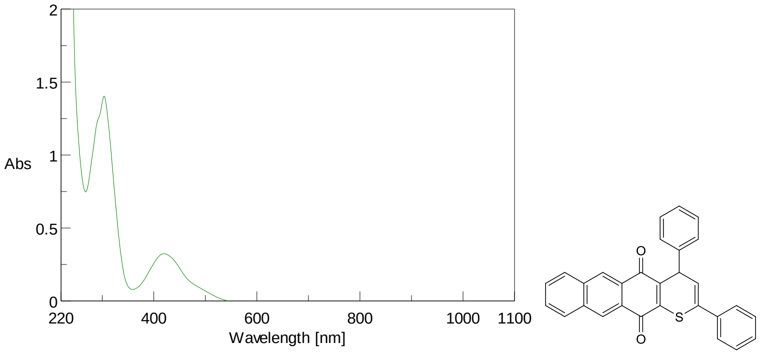

Figure S16: The UV-VIS spectrum for 4e

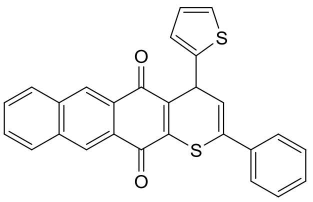

4f

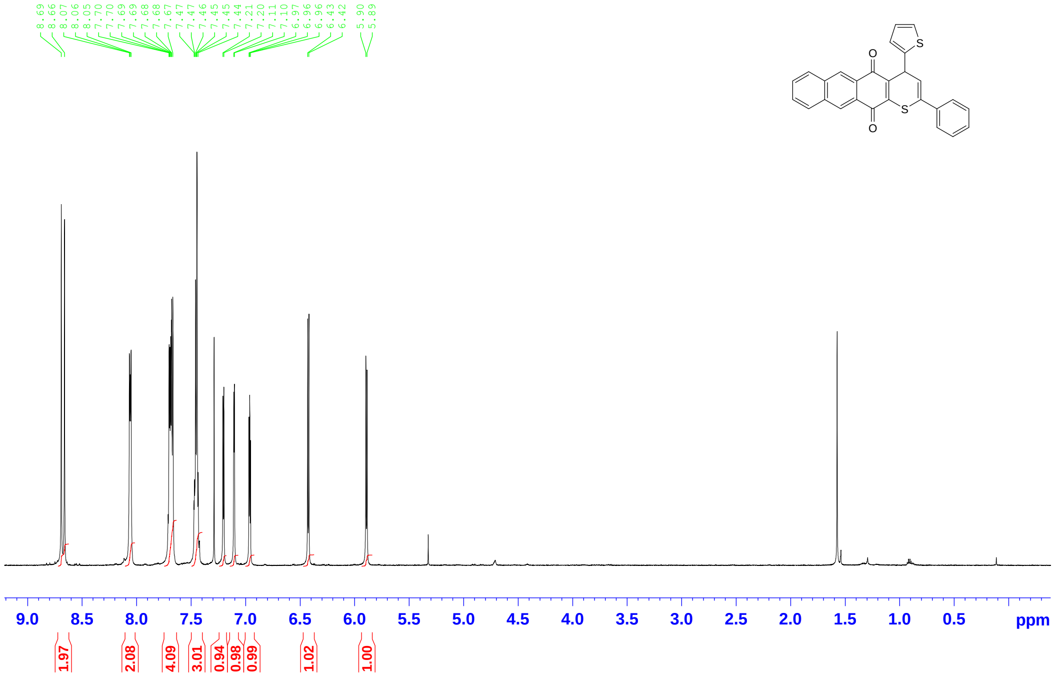

Figure S17: The ^{1}H NMR spectrum for 4f

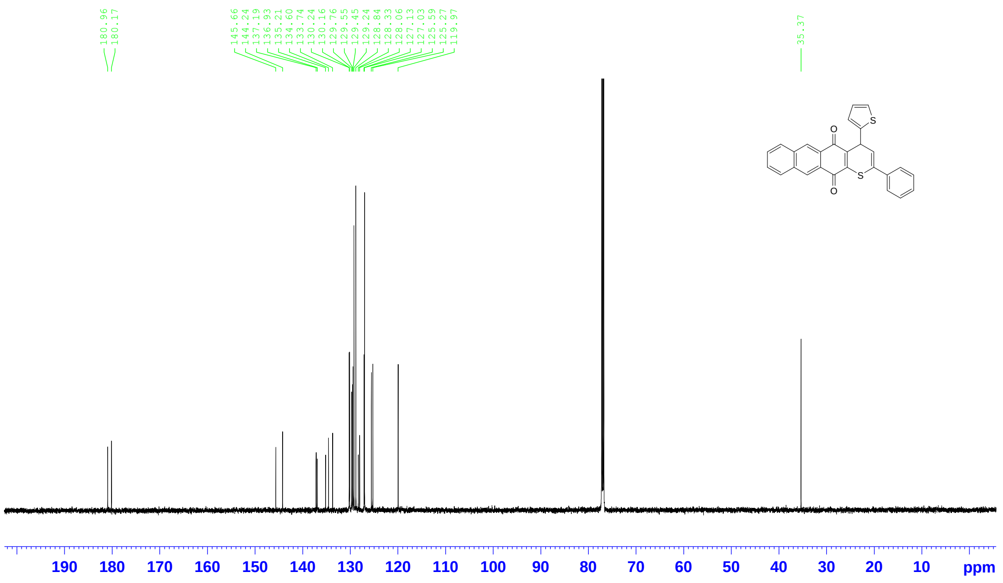

Figure S18: The ^{13}C NMR spectrum for 4f

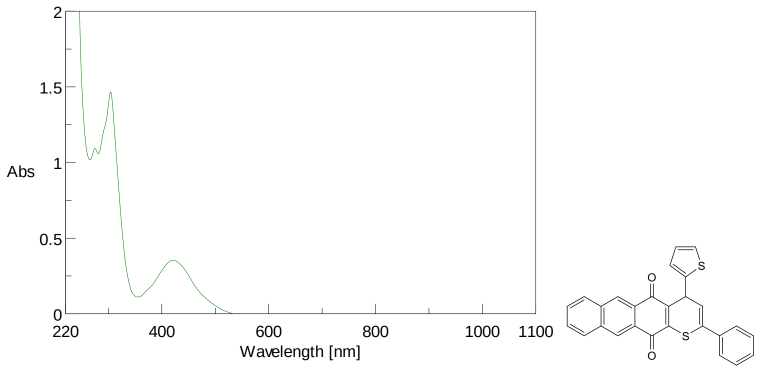

Figure S19: The UV-VIS spectrum for 4f

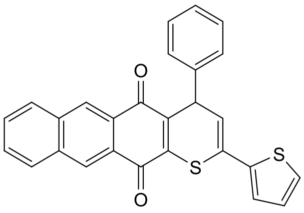

4g

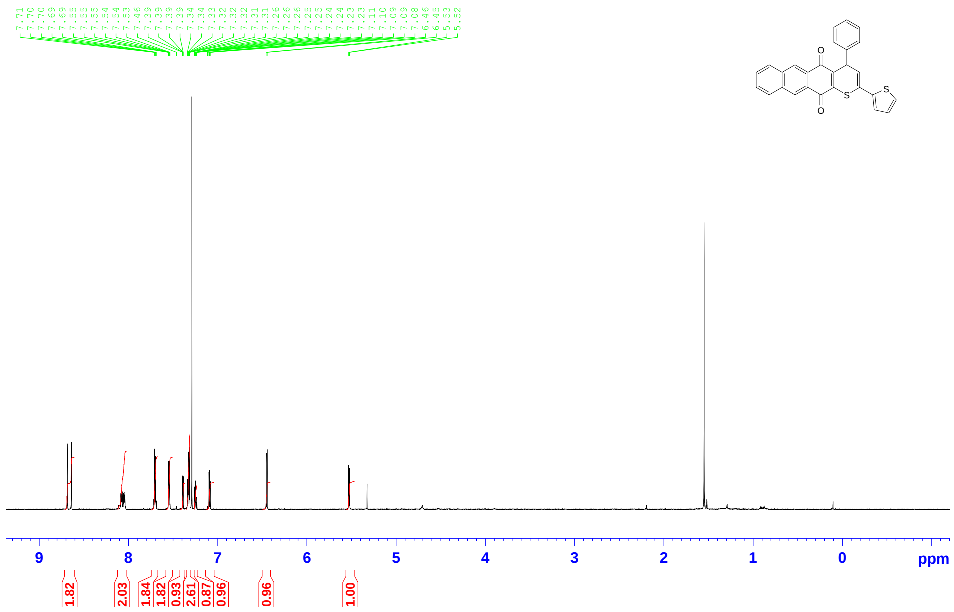

Figure S20: The ^{1}H NMR spectrum for 4g

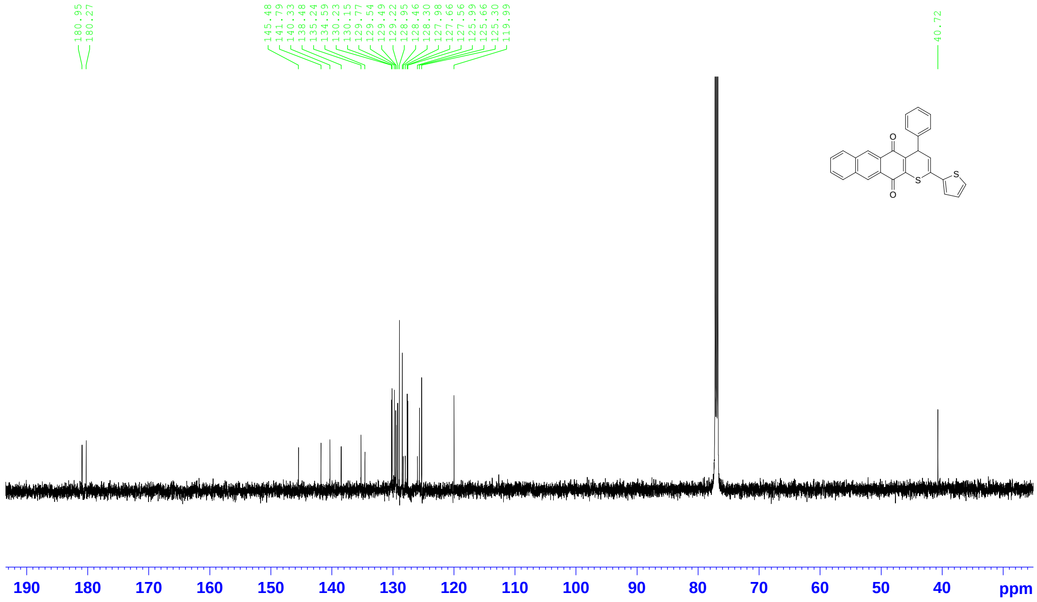

Figure S21: The ^{13}C NMR spectrum for 4g

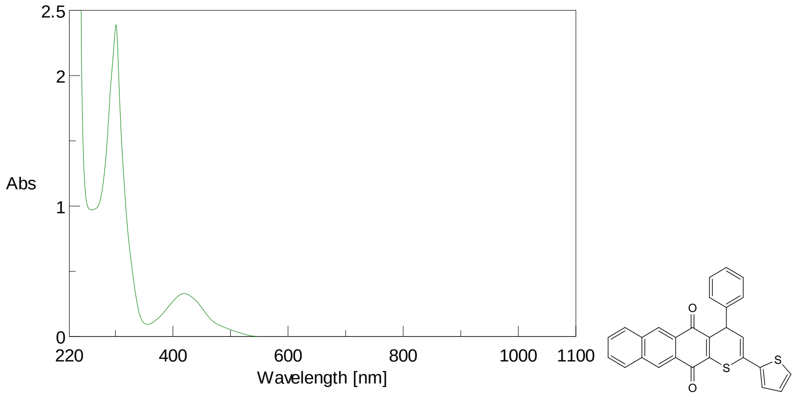

Figure S22: The UV-VIS spectrum for 4g

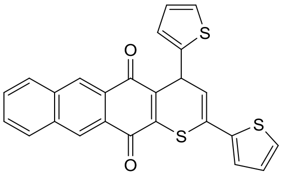

4h

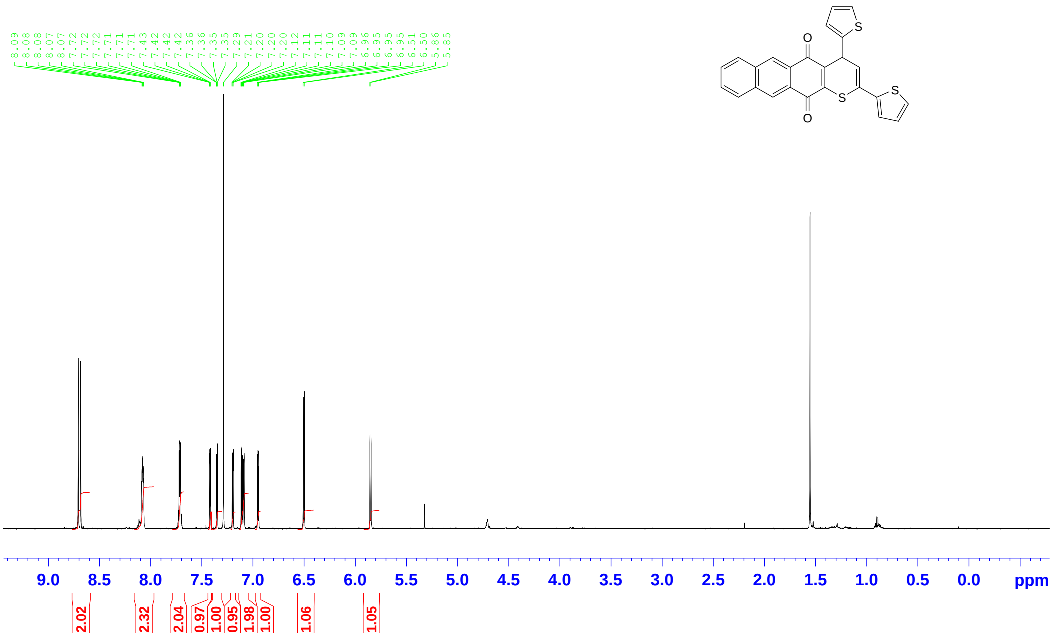

Figure S23: The ^{1}H NMR spectrum for 4h

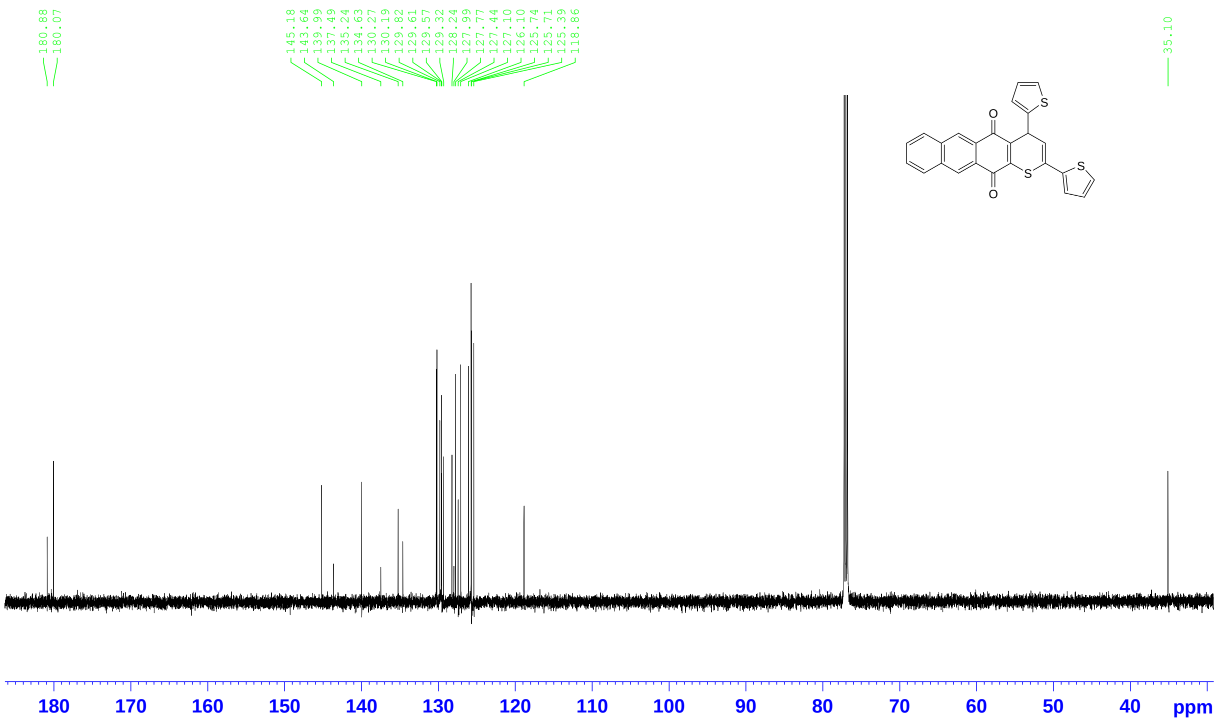

Figure S24: The ^{13}C NMR spectrum for 4h

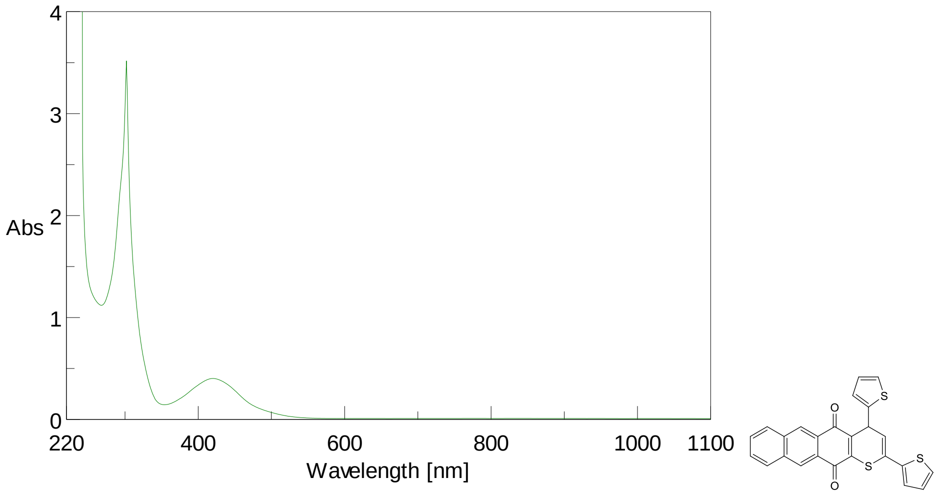

Figure S25: The UV-VIS spectrum for 4h

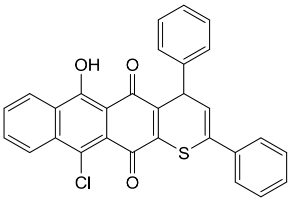

4i

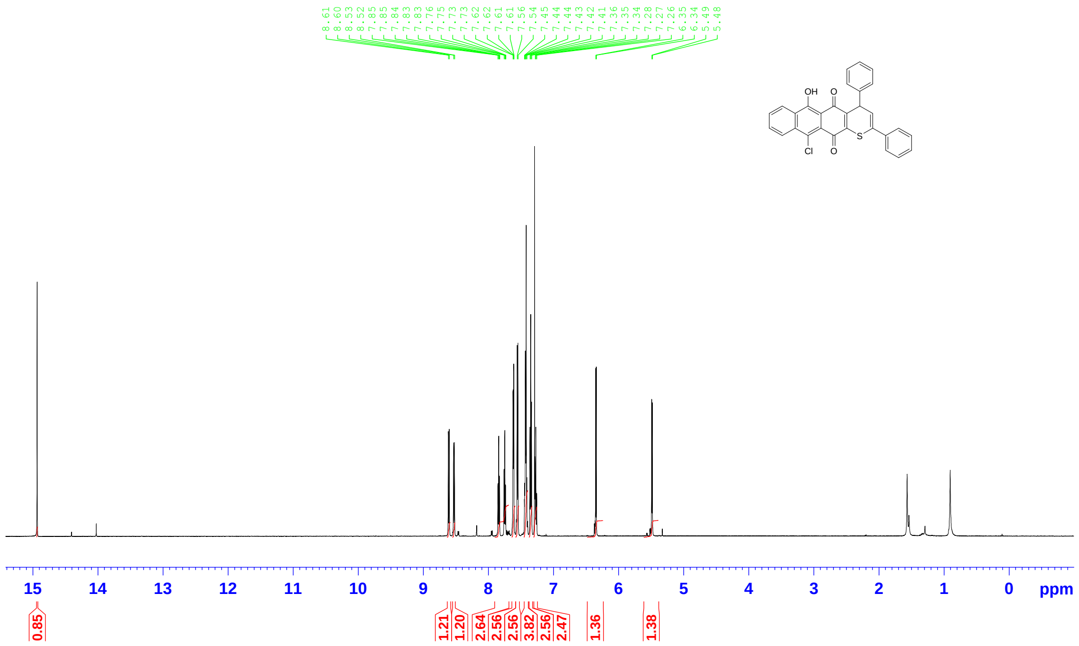

Figure S26: The ^{1}H NMR spectrum for 4i

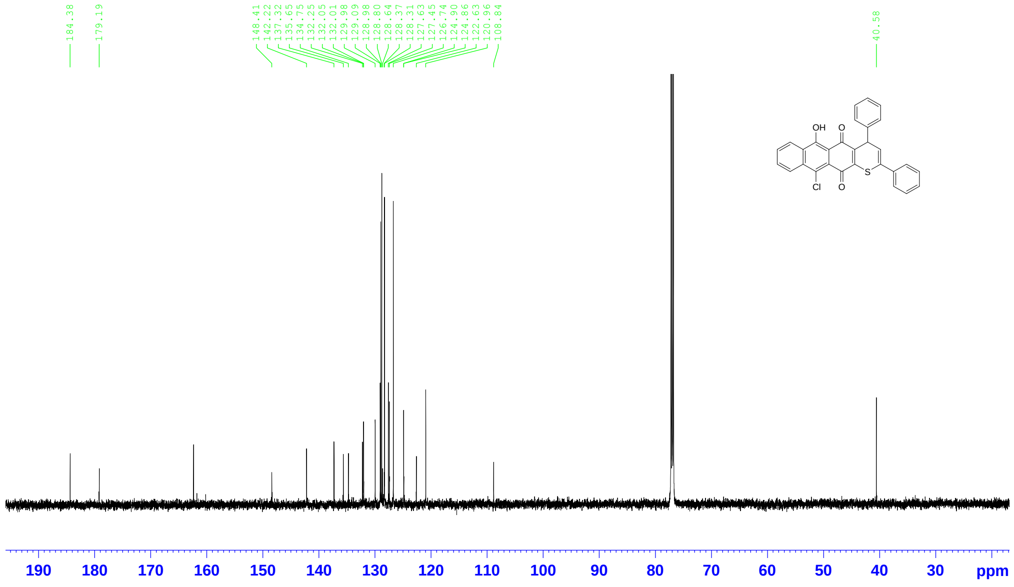

Figure S27: The ^{13}C NMR spectrum for 4i

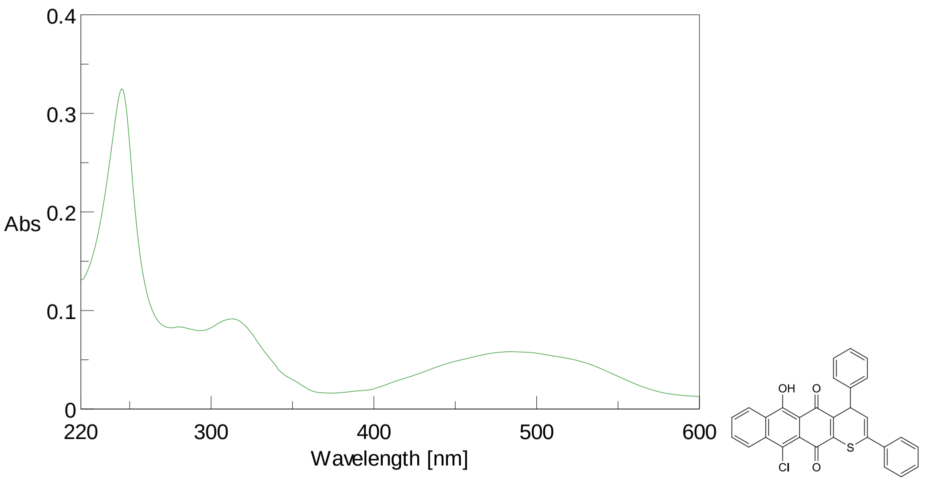

Figure S28: The UV-VIS spectrum for 4i

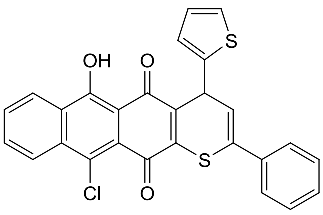

4j

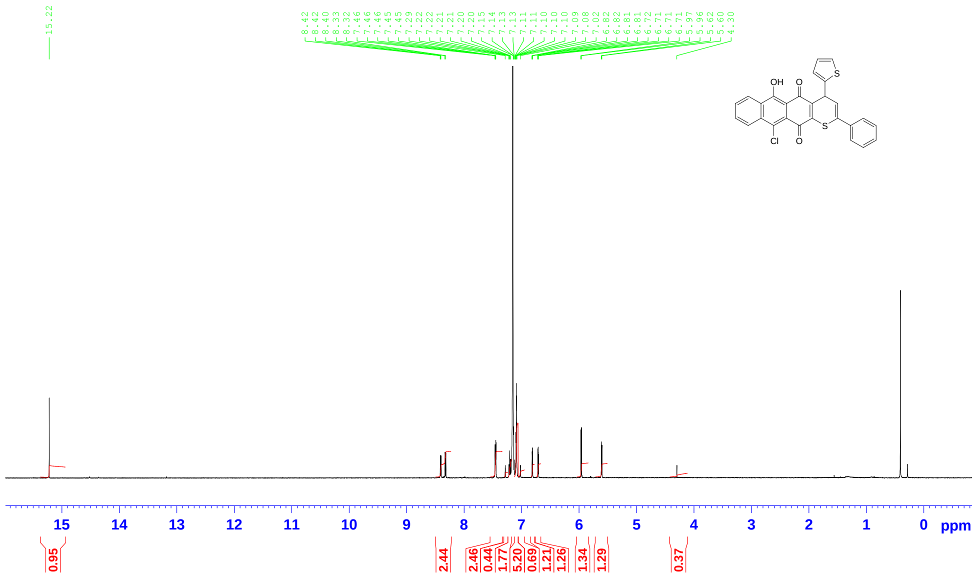

Figure S29: The ^{1}H NMR spectrum for 4j

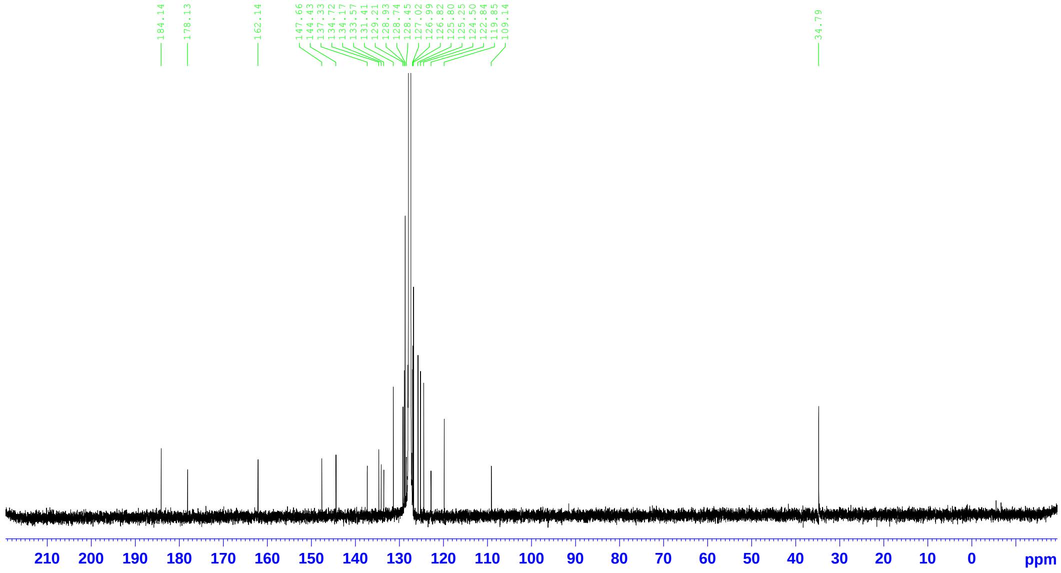

Figure S30: The ^{13}C NMR spectrum for 4j

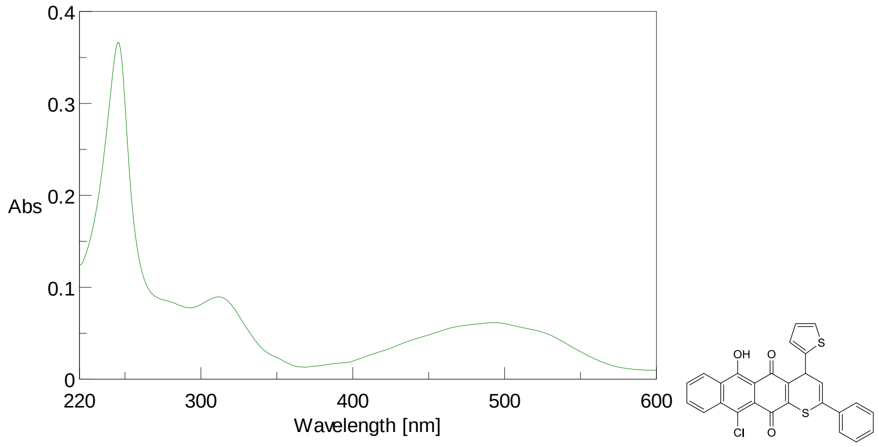

Figure S31: The UV-VIS spectrum for 4j

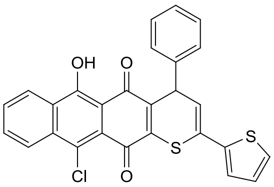

4k

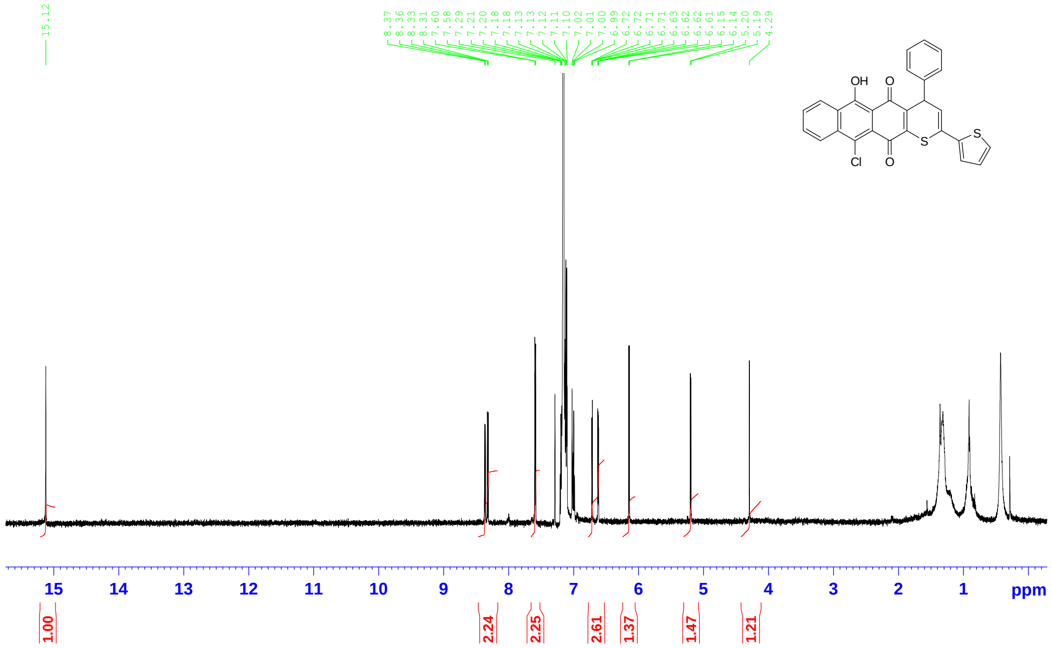

Figure S32: The ^{1}H NMR spectrum for 4k

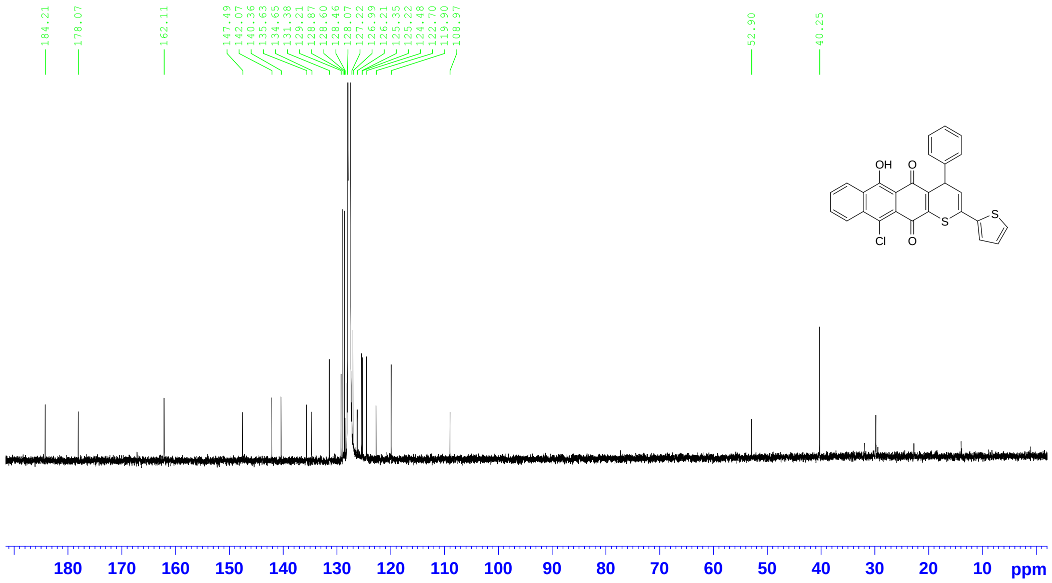

Figure S33: The ^{13}C NMR spectrum for 4k

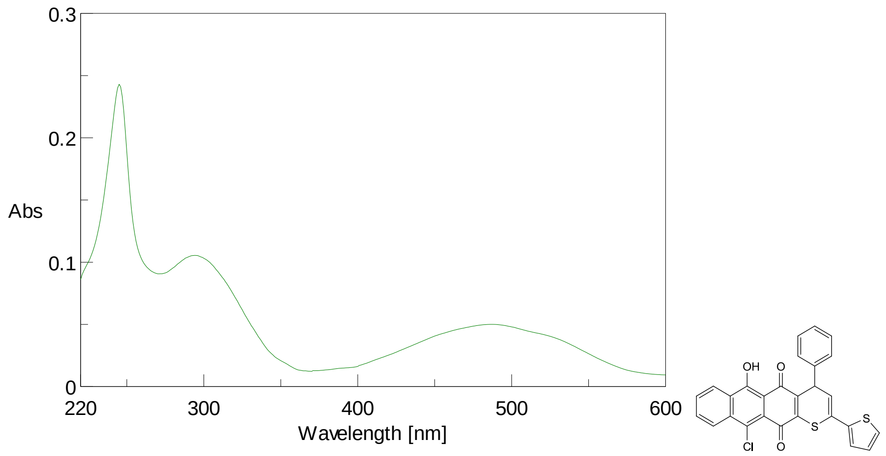

Figure S34: The UV-VIS spectrum for 4k

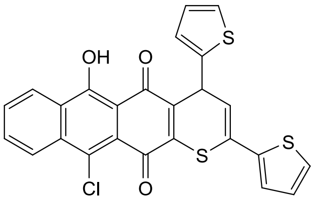

4l

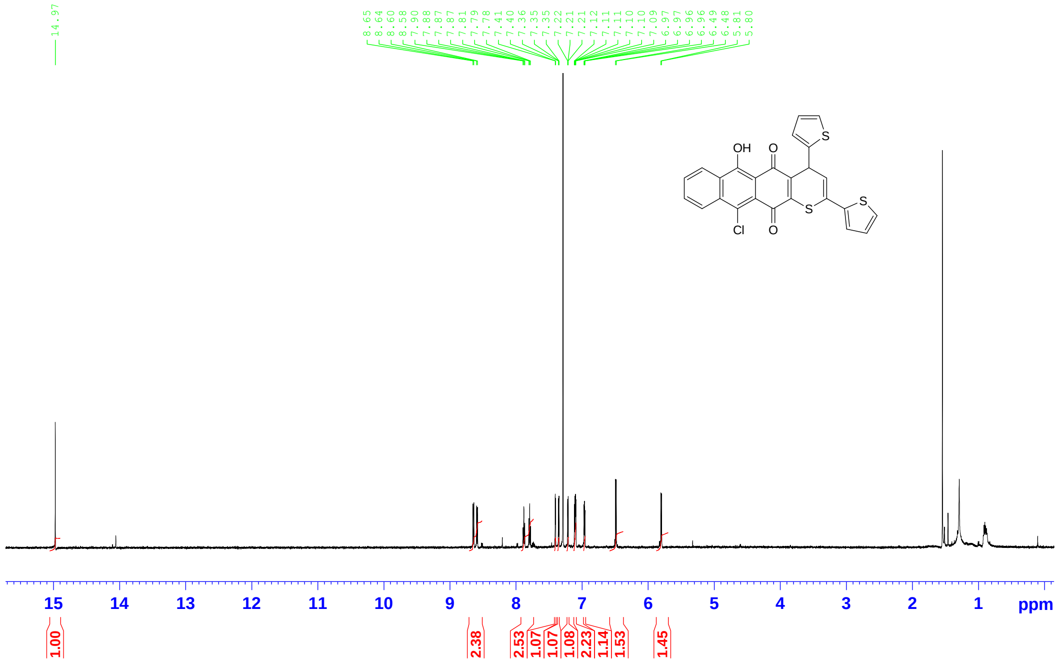

Figure S35: The ¹H NMR spectrum for 4l

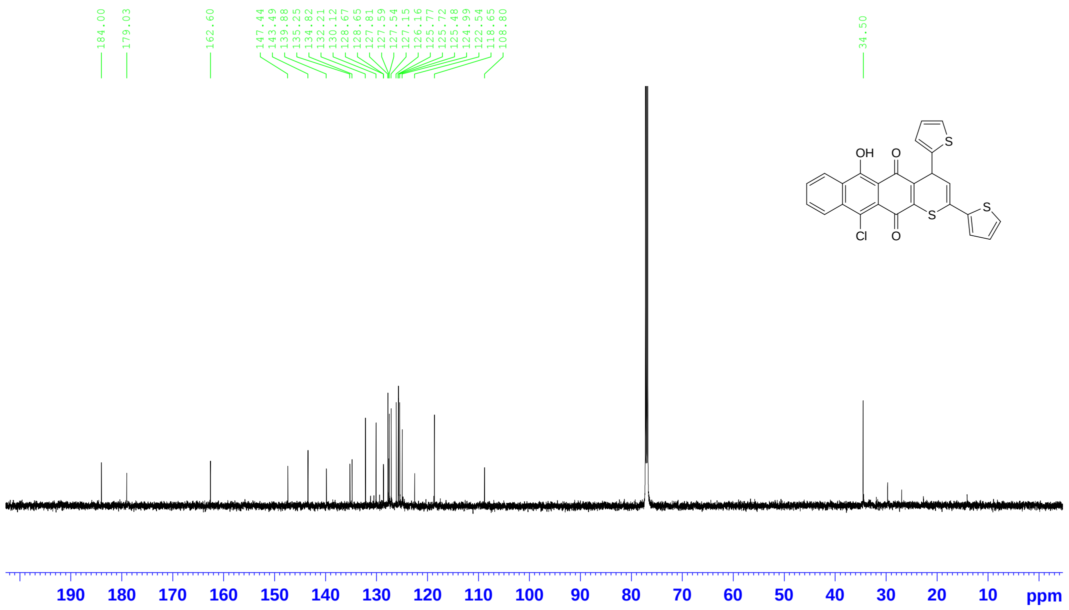

Figure S36: The ^{13}C NMR spectrum for 4l

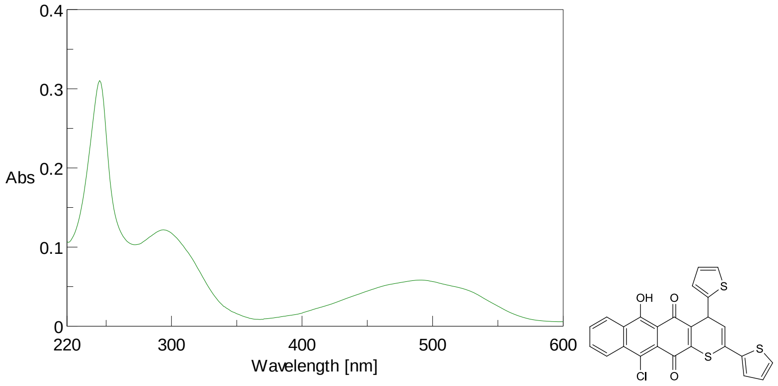

Figure S37: The UV-VIS spectrum for 4l

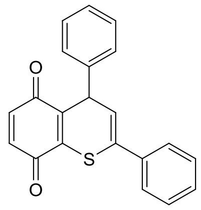

4m

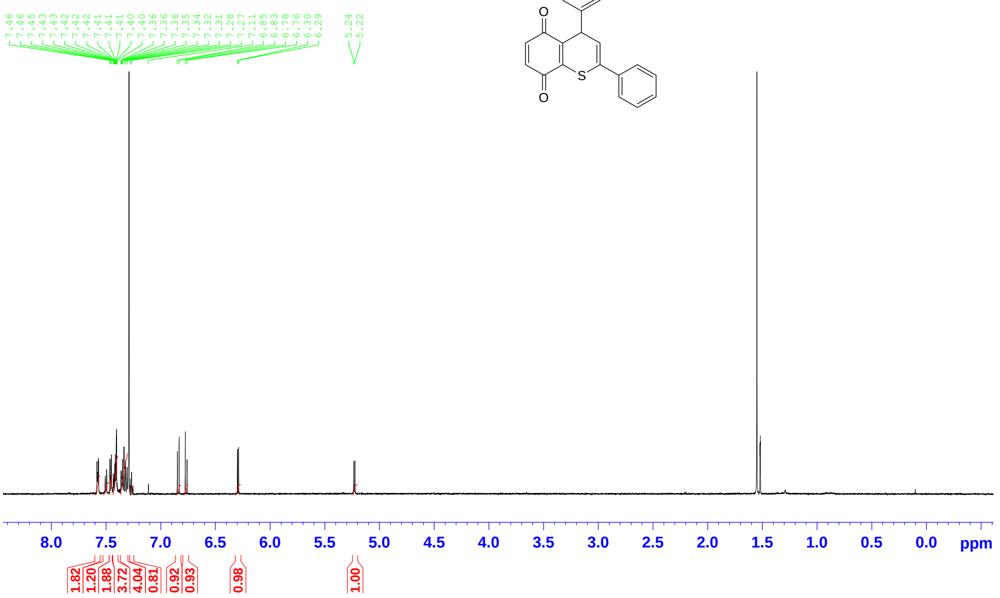

Figure S38: The ^{1}H NMR spectrum for 4m

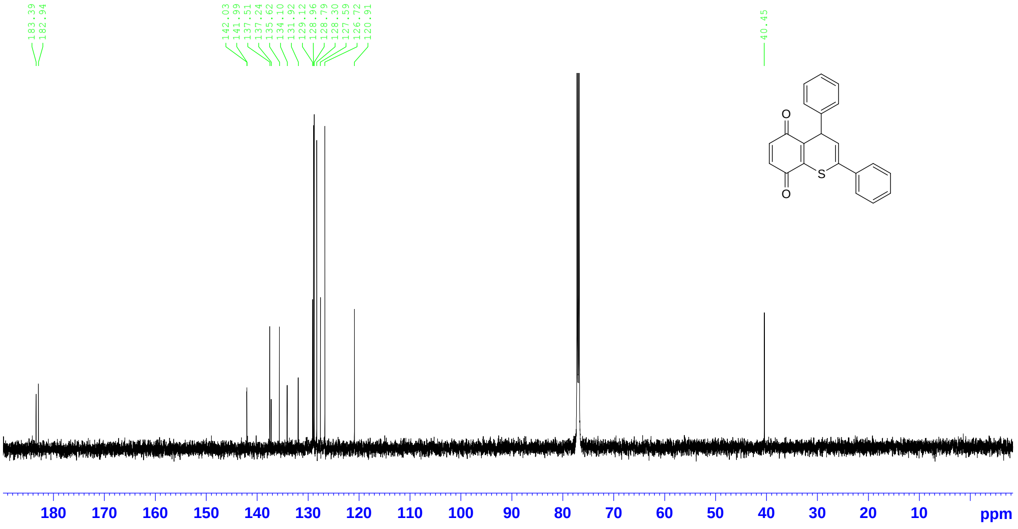

Figure S39: The ^{13}C NMR spectrum for 4m

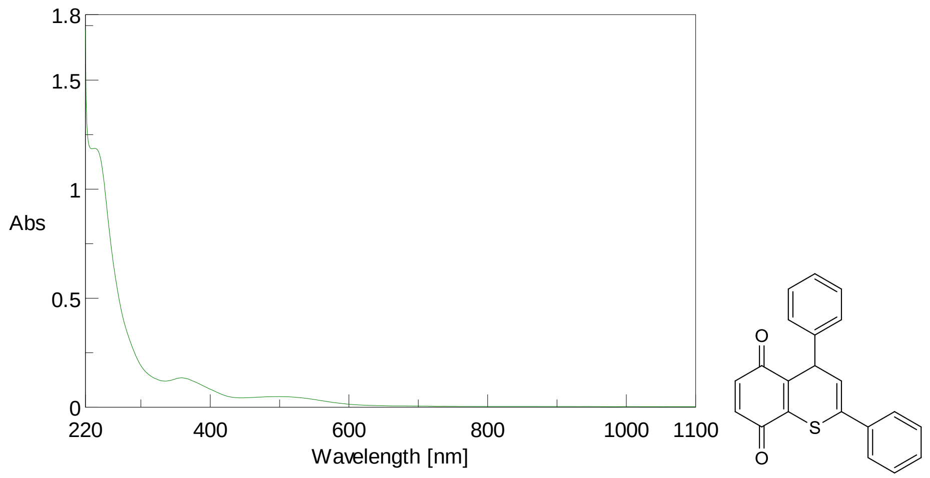

Figure S40. UV-VIS spectrum for 4m

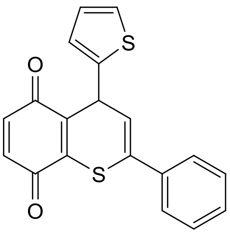

4n

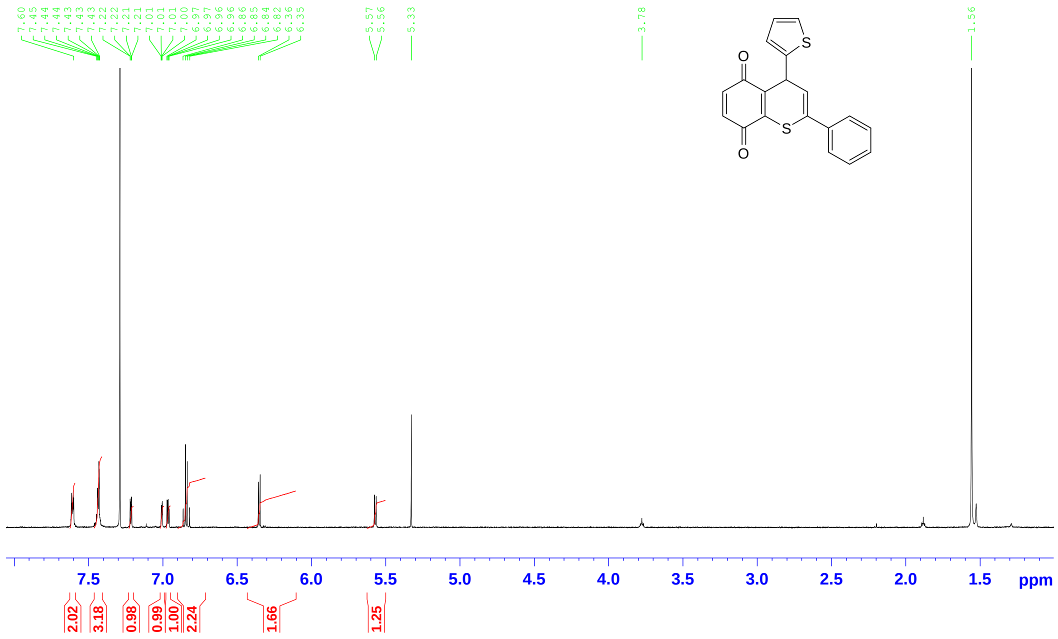

Figure S41: The ^{1}H NMR spectrum for 4n

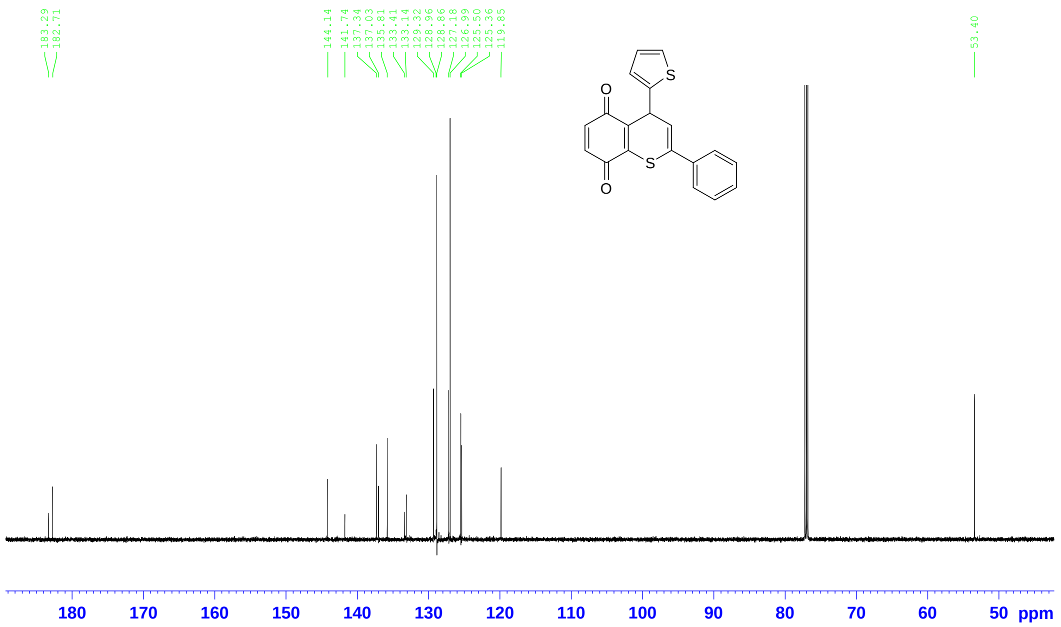

Figure S42: The ^{13}C NMR spectrum for 4n

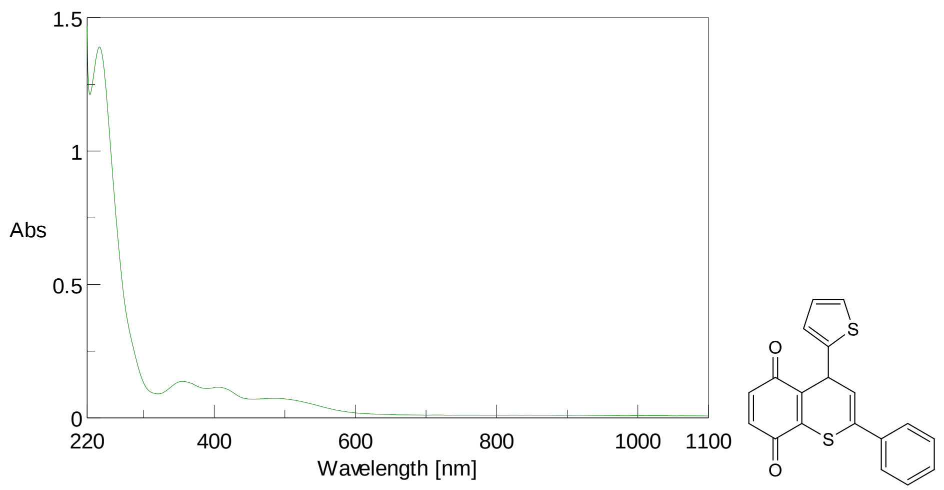

Figure S43: The UV-VIS spectrum for 4n

The ^{1}H and ^{13}C NMR spectra for the cycloadduct 6

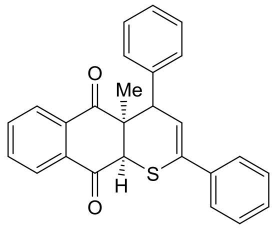

6

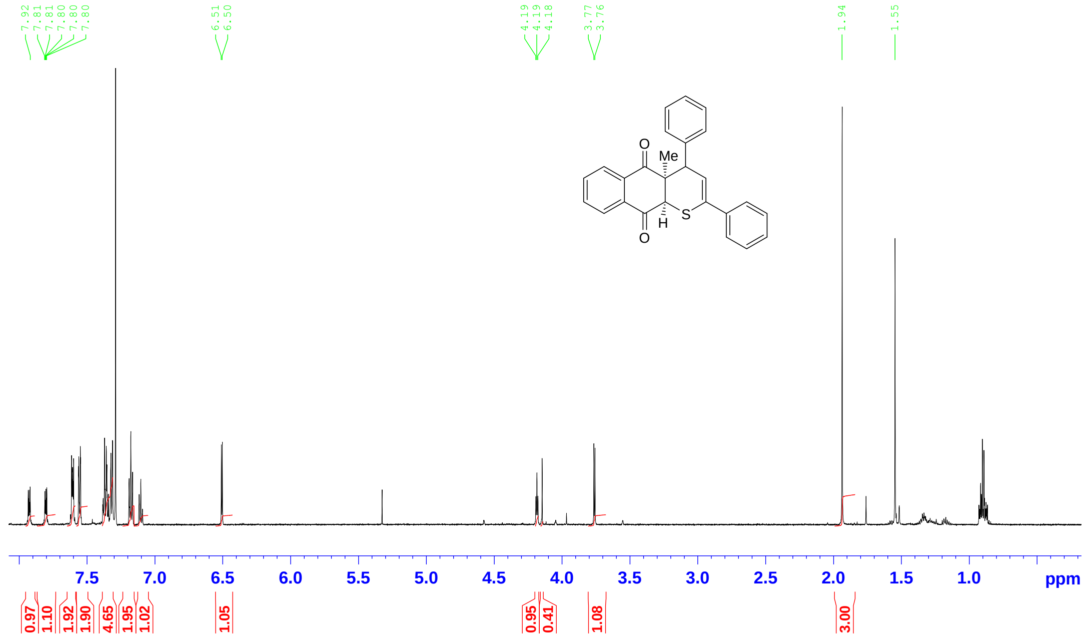

Figure S44: The ^{1}H NMR spectrum for 6

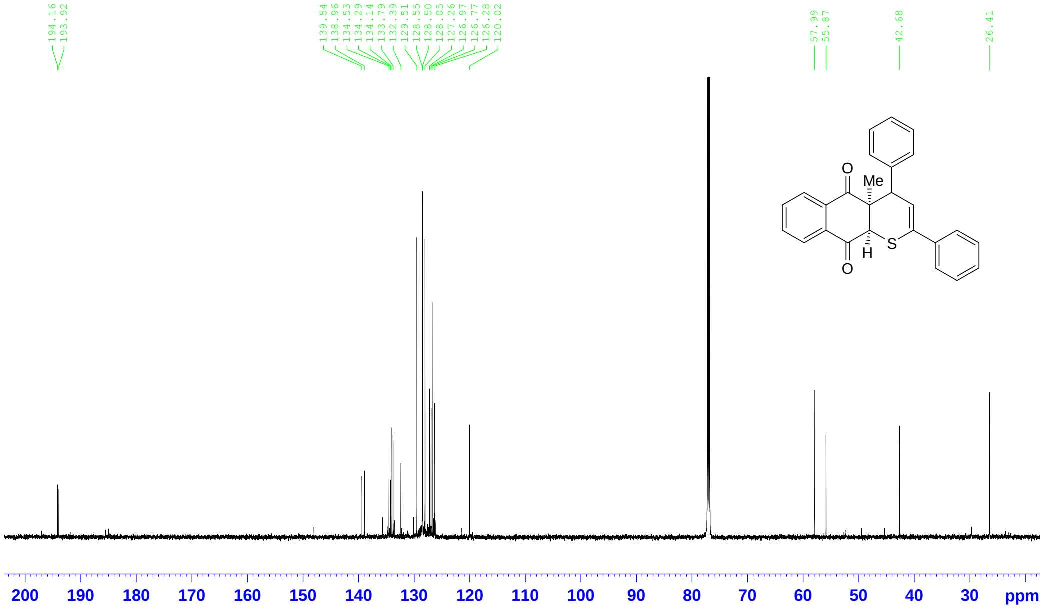

Figure S45: The ^{13}C NMR spectrum for 6
